# A bioinspired hollow g-C_3_N_4_–CuPc heterostructure with remarkable SERS enhancement and photosynthesis-mimicking properties for theranostic applications†

**DOI:** 10.1039/d2sc01534j

**Published:** 2022-05-12

**Authors:** Yu Su, Baozhen Yuan, Yaowen Jiang, Ping Wu, Xiaolin Huang, Jun-Jie Zhu, Li-Ping Jiang

**Affiliations:** State Key Laboratory of Analytical Chemistry for Life Science, School of Chemistry and Chemical Engineering, Nanjing University Nanjing Jiangsu 210023 China; State Key Laboratory of Food Science and Technology, School of Food Science and Technology, Nanchang University Nanchang 330047 China; Jiangsu Key Laboratory of New Power Batteries, College of Chemistry & Materials Science, Nanjing Normal University Nanjing Jiangsu 210097 China; Shenzhen Research Institute of Nanjing University Shenzhen 518000 China

## Abstract

Surface-enhanced Raman scattering (SERS) based on chemical mechanism (CM) has great potential for superior stability and selectivity. Moreover, a bioinspired CM-Raman substrate-Raman reporter system with charge separation and electron transport nature provides thylakoid-mimicking potential for multifunctional applications. Herein, hollow carbon nitride nanospheres hierarchically assembled with a well-oriented copper(ii) phthalocyanine layer and hyaluronic acid (HCNs@CuPc@HA) were designed as a light-harvesting nanocomposite and photosynthesis-mimicking nanoscaffold that enhance both CM-SERS and photoredox catalysis. Remarkable SERS enhancement was achieved due to the strengthened short-range substrate–molecule interaction, enriched CuPc molecule loading and enhanced light–mater interactions. Meanwhile, the uniform CuPc molecule film mimics a photo-pigment to accelerate the near infrared (NIR)-oxygen generation and photodynamic catalysis of photosynthetic membrane-like HCNs. The experimental findings were further validated by numerical theory analysis. The greatly enhanced SERS signal and photosynthetic-mimicking properties of the heterostructure (denoted as HCNCHs) were successfully employed for circulating tumor cell (CTC) diagnosis and SERS imaging-guided cancer catalytic therapy in tumor xenograft models.

## Abstract 2

Thylakoid-inspired HCNs@CuPc@HA is designed as a light-harvesting nanocomposite and photosynthesis-mimicking nanoscaffold to simultaneously enhance chemical mechanism-based SERS and photosynthesis-mimicking catalysis for theranostics application.

## Graphical abstract

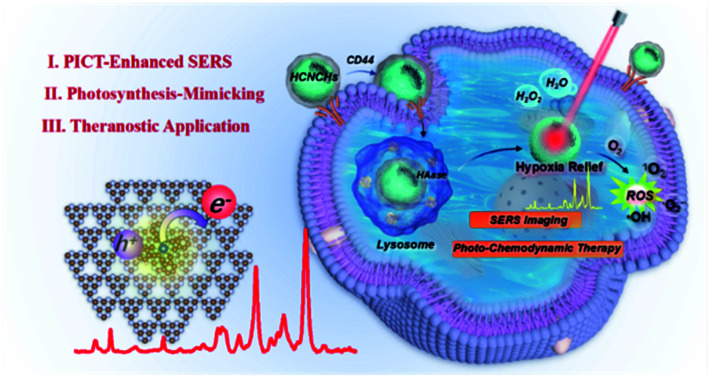

## Introduction

Surface-enhanced Raman scattering (SERS) has been an emerging analytical technique in the field of sensing and imaging.^1,2^ It provides striking advantages of good sensitivity, high spectral resolution, superior resistance to photo-bleaching or auto-fluorescence with noninvasive sampling.^3,4^ Besides plasmonic-based SERS,^5^ the chemical enhancement mechanism (CM)-enhanced Raman signal exhibits higher uniformity and selectivity, especially independent of agglomeration.^6–8^ Especially, the CM is related to the electronic transitions between the adsorbed Raman molecule and enhancing substrate. Nonmetallic SERS nanoplatforms based on the chemical enhancement of graphene, hexagonal boron nitride and semiconducting metal oxides have been developed to detect biomolecules, such as DNA, RNA, protein, and so on.^9–13^ However, the short-range interaction between the CM-substrate and molecules calls for more efficient interfacial interactions to lift the actual enhancement factor (EF).^14^ Moreover, analogous to rough plasmonic Raman enhancing substrates to concentrate an optical energy field, the photoinduced charge transfer (PICT) dependent CM-SERS enhancing process is largely related to the substrate/molecule-light interactions.^15^ Compared to solid nanomaterials, hollow porous nanoparticles are expected to exhibit more enhanced interaction with light and surrounding media for more efficient charge transfer (CT).^16,17^

Recently, hollow nanostructured materials mimicking vesicles and cells have also attracted attention because of the enhanced photocatalysis effect.^18,19^ In combination with photosensitive Raman reporters such as metallophthalocyanine (MPc) acting as a photosynthetic pigment grafted on the surface, the CM-SERS substrate–molecule system is prominent to mimic natural photosynthesis.^20^ Similar to the photosynthetic thylakoid membrane incorporated with a water-oxidation centre and carbon dioxide-reduction centre, efficient energy transfer and photon conversions in a nanosized substrate–molecule biomimetic system could proceed for optical and catalytic performances.^21^ Particularly, inspired by thylakoids, a hollow organic semiconductor is a promising optical nanogenerator allowing for the direct generation of reactive electrons and holes on the shell with sufficient energy for redox chemistry.^22,23^ In this way, the water splitting reaction and photodynamic catalysis could be simultaneously initiated for oxygen (O_2_) and reactive oxygen species (ROS) generation. Therefore, the “intelligent” form of a CM-SERS heterostructure as a bright SERS contrast agent and catalytic nanoagent for cancer theranostics is of great interest and practical value.

**Scheme 1:**
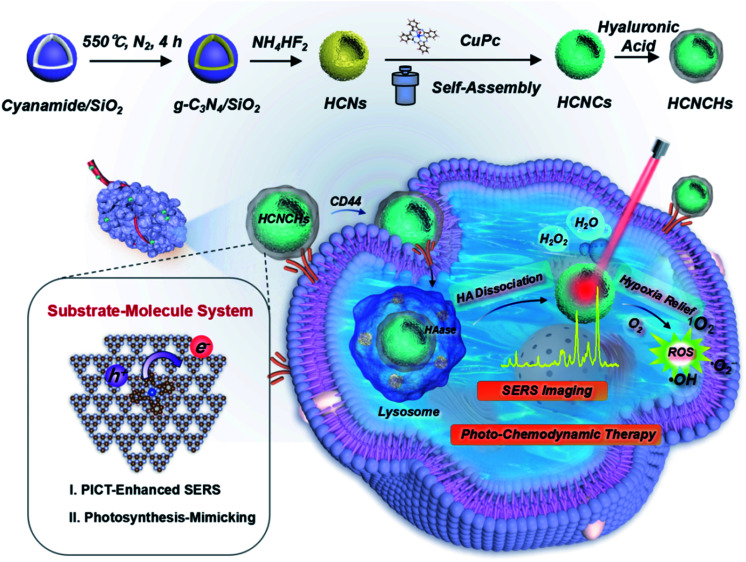
Schematic illustration of the bioinspired HCNCHs and theranostic platform featuring CM-SERS diagnosis, and photosynthetic-mimicking performance-enhanced photo-chemodynamic therapy.

In this study, an integrate platform based on polymeric hollow nanospheres of graphitic carbon nitride (g-C_3_N_4_) hierarchically assembled with well-oriented CuPc and HA is designed and proposed to enhance CM-SERS and photocatalysis for cancer theranostics (Scheme 1). Light-harvesting hollow carbon nitride nanospheres (HCNs) adhered with a uniform CuPc molecule film (HCNs@CuPc, HCNCs) mimic a photo-pigment modified photosynthetic membrane for fast charge separation and electron transport. Thus, excellent CM-SERS enhancement was achieved from the efficient PICT process. Meanwhile, the PICT line contributes to the photosynthetic-mimicking performance of an enhanced NIR oxygen evolution reaction (nOER) and photodynamic catalysis. Moreover, the designed hybrid nanocomposite was demonstrated as a peroxidase-mimicking (POD) enzyme that catalyzes H_2_O_2_ conversion into reactive ˙OH. The experimental findings were further validated by numerical theory analysis. Finally, the functional heterostructure was used as a SERS detective nanoprobe and therapeutic nanocatalyst for biomedical applications. Upon CD44 receptor-mediated internalization and hyaluronidase (HAase)-responsive catalytic surface exposure in a tumor microenvironment (TME), tumor-specific phototheranostics was achieved through *in vitro* and *in vivo* SERS diagnosis and imaging-guided hypoxic TME-modulated/responsive synergistic photo-chemodynamic therapy with an excellent outcome. We believe that the proposed bioinspired SERS substrate–molecule system warrants widespread biomedical applications for diagnosis, imaging, and disease treatment.

## Results and discussion

### Synthesis and characterization of the HCNCs and HCNCHs

**Fig. 1:**
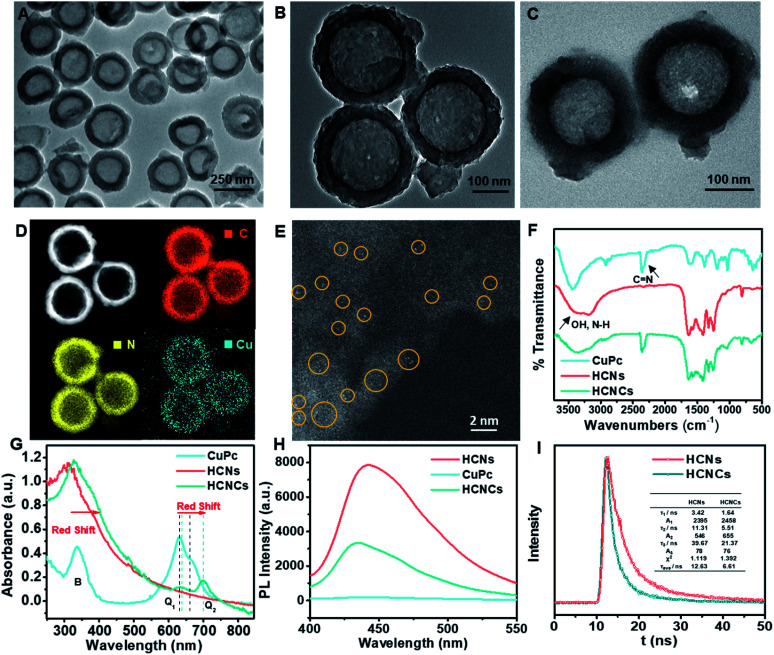
(A and B) TEM images of HCNCs. (C) TEM image of HCNCHs. (D) EDX elemental mapping images of C, N and Cu on HCNCs. (E) Typical view of the HAADF-STEM image of distributed single Cu atoms on HCNCs. Yellow circles indicate typical single Cu atoms. (F) FT-IR, (G) UV-vis and (H) steady-state PL spectra of CuPc, HCNs and HCNCs. (I) PL decay curves of HCNs and HCNCs.

The synthesis of the HCNC composite involved one-pot solvothermal assembly using the HCNs as the core substrate. The HCNs with a hollow vesicle polymer geometry (Fig. S1B†) were synthesized using mesoporous silica nanospheres (Fig. S1A†) as a sacrificial template. Then, a confined CuPc molecular film was deposited on the HCNs to form HCNCs with a blue color (Fig. S2†) through solvothermal-enhanced π–π interaction.^24,25^ The transmission electron microscopy (TEM) image (Fig. 1A and B) and high resolution TEM (HRTEM) images (Fig. S3†) show that the HCNCs maintain the hollow spherical morphology and mesoporous structure of HCNs after the hydrothermal reaction and are well monodispersed with a uniform size of ∼250 nm. Thermogravimetric analysis (TGA) demonstrates the excellent thermal stability of the HCNCs (Fig. S4†). After HA modification, the mesoporous structure was clouded with a sticky HA layer (Fig. 1C). Zeta potential and average hydrodynamic diameter results confirm the successful HA encapsulation (Fig. S5†). Moreover, the HCNCHs demonstrate excellent stability in different media during 4 weeks of incubation (Fig. S6†). Further energy dispersive X-ray (EDX) elemental mapping images display the uniform distribution of C, N, and Cu on the HCNCs, implying the successful assembly of CuPc on the HCNs (Fig. 1D). The high-angle annular dark-field scanning transmission electron microscopy (HAADF-STEM) image in Fig. 1E reveals the distribution of Cu atoms on HCNCs and directly confirms the molecular-level dispersion of CuPc.^26^ Brunauer–Emmett–Teller (BET) analysis suggests that the mesoporous nanostructure of HCNs greatly lifts the molecule loading capacity (Fig. S7†). X-ray photoelectron spectra (XPS) (Fig. S8†) also indicate the successful loading of CuPc. X-ray diffraction (XRD) analysis in Fig. S9† demonstrates that the HCNCs present two typical peaks at 12.9° and 27.7° identical to HCNs, which correspond to the (100) and (002) planes of g-C_3_N_4_, originating from graphitic-like layered stacking of CN-based materials.^27^ Moreover, a weak peak appears at 22.7°, which could be accounted for by the interlayer stacking between the HCNs and CuPc system, indicating heterojunction formation.^28^ The Fourier transform infrared (FT-IR) spectra in Fig. 1F suggest that the successful hybridization between HCNs and CuPc is reinforced by H-bond interactions.^29,30^ UV-vis absorption spectroscopy was also used to investigate the binding mode between HCNs and CuPc. As shown in Fig. 1G, CuPc presents a phthalocyanine-characteristic Soret (B) band at 336 nm, as well as a Q band at 630 nm (Q1) and 663 nm (Q2), which are favorable for visible/NIR light excitation. The Q band is closely related to the π–π* transition between bonding and antibonding molecular orbitals, in which the intense Q1 band is attributed to the aggregated CuPc molecules due to the intermolecular face-to-face stacking and the weaker Q2 shoulder band is assigned to the monomeric species.^31^ The HCNs display the typical absorption onset of g-C_3_N_4_ at 420 nm, corresponding to its band gap of 2.7 eV. In contrast, the absorption band of HCNCs exhibits the obvious appearance of the Q band of CuPc in the NIR light region, indicating the combination of the two species. Interestingly, the relative intensity of the Q1 and Q2 bands of HCNCs involves an inverse transform compared to that of CuPc, which is similar to reported graphene-enhanced Raman scattering (GERS).^32^ This demonstrates that the proportion of the CuPc aggregates and monomers becomes smaller and larger respectively, and the CuPc molecules tend to lie down on the surface of HCNs, which implies the uniform orientation of the molecule layer.^32^ Moreover, the Q band of HCNCs is apparently red-shifted by 8 nm for Q1 and 37 nm for Q2, suggesting a decreased π–π* transition energy due to the strong coupling of the two species.^32^ Furthermore, the migration, transfer and exciton recombination dynamics of photo-generated electron–hole pairs of HCNCs were investigated by steady-state photoluminescence (PL) spectroscopy and time-resolved PL. As shown in Fig. 1H, the PL intensity of HCNCs is significantly lower than that of HCNs with an obvious blue shift of 7 nm due to quantum confinement caused by the stacking of the CuPc layer.^33^ The shorter PL lifetime (Fig. 1I) indicates fast luminescence quenching of HCNCs, which might be attributed to the fact that charge separation is enhanced by extended π-conjugated systems and delocalization of the π-electrons after the CuPc conjugation.^34^ This suggests additional decay pathways for the photoexcitation, by which the CT process is expected to improve the SERS performance and photosynthesis-mimicking activity.

### CM-SERS, photosynthesis-mimicking and peroxidase-mimicking enhancements on HCNCs

**Fig. 2:**
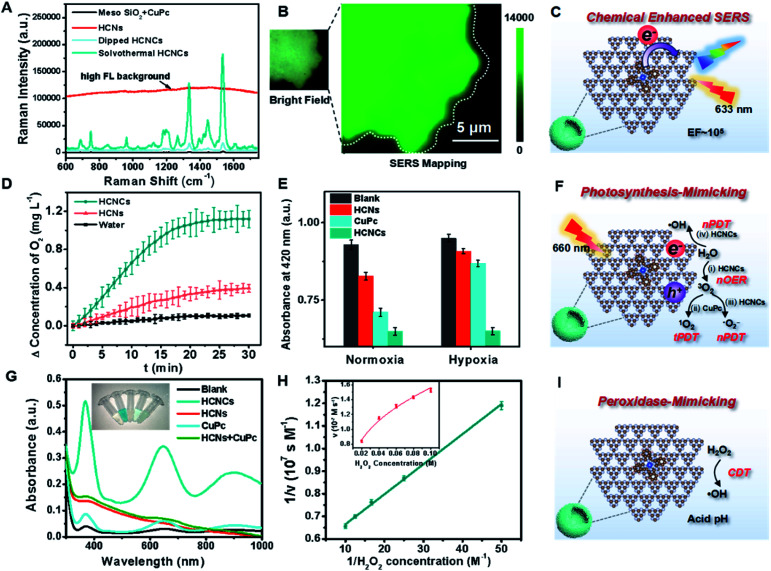
(A) SERS spectra of mesoporous silica nanospheres + CuPc, HCNs, dipped HCNCs and solvothermal HCNCs in the solid state, with a 633 nm laser, 1.7 mW power and 10 s exposure time. (B) The bright field and Raman mapping image for the vibrational mode of HCNCs at 1533 cm^−1^ with a 633 nm laser, 1.7 mW power and 1 s exposure time. (D) O_2_ generation curves of water, HCNs and HCNCs under light irradiation (660 nm, 0.282 W cm^−2^) within 30 min; (E) ROS generation efficiency of water, HCNs, CuPc and HCNCs in normoxic and hypoxia environments under light irradiation (660 nm, 0.282 W cm^−2^) for 10 min. (G) UV-vis absorption spectra of oxTMB catalyzed by water, HCNCs, HCNs, CuPc and HCNs + CuPc. The inset shows the corresponding visual color changes. (H) Kinetics for the POD activity of HCNCs. (C, F and I) Schematic illustration of HCNC-enhanced CM-SERS, photosynthesis-mimicking and peroxidase-mimicking performances.

The surface/interface-related performances of HCNCs, including SERS, photosynthesis-mimicking and POD properties, were systematically investigated. As shown in Fig. 2A, the bare HCNs have no obvious Raman characteristic peak but exhibit an extremely high fluorescence (FL) background signal exceeding 100 000 in the whole spectral range. The mixture of a mesoporous silica template and CuPc exhibits a weak SERS signal. Encouragingly, the SERS signal of solvothermal HCNCs (100 μg mL^−1^) shows a disappeared FL background and reaches a prominent level of ∼180 000 at 1533 cm^−1^. This suggests that the combination of CuPc effectively quenches the intrinsic FL signal of HCNs, and the HCNs substrate is an excellent candidate for CM-SERS. More importantly, the solvothermal HCNCs exhibit an order of magnitude higher signal intensity compared to the dipped HCNCs prepared by the impregnation method, which is attributed to the increased molecule loading amount of the “substrate–molecule” system through strengthening the π–π and H-bond interactions (Fig. S10†). Benefiting from the uniform CuPc molecular layer growth, the SERS mapping of the intensity at 1533 cm^−1^ from HCNCs shows a homogeneous distribution (Fig. 2B). Also, compared to g-C_3_N_4_ nanosheets, the mesoporous hollow nanostructure is preferable for SERS enhancement because of its molecule loading and unique light-harvesting advantages (Fig. S11†). We also found the same SERS signal intensity from the freshly prepared HCNCs upon 28 days of storage, implying the stable adsorption of CuPc onto HCNs (Fig. S12†). The synthetic temperature (*T*) was optimized to be 120 °C with comparatively high SERS intensity and particle monodispersity for further bioapplications (Fig. S13–S15†). The influences of the excitation laser wavelength and power on SERS performance were also studied (Fig. S16†). Moreover, the solution-state SERS intensity of HCNCs remains at a high level of ∼30 000 at 1533 cm^−1^ (Fig. S17†), which indicates that the conjugate is expected to break through the limitations of traditional solid phase detection in CM materials. The EF was calculated to be 1.84 × 10^5^, which approaches the general level of plasmonic materials and is highly competitive among the existing CM-based SERS nanoprobes.^35,36^ Furthermore, the SERS performance of HCNCHs is maintained after HA modification and HAase-mediated HA dissociation^37^ (Fig. S19†) and stable in different media (Fig. S20†).

Subsequently, the photosynthesis-mimicking nOER and tandem PDT (tPDT) performances of HCNCs were evaluated. As shown in Fig. 2D, the HCNC-dispersed solution exhibits an O_2_-evolving concentration of 3 times higher than that of HCNs, indicating that the CuPc conjugation on HCNs could efficiently improve the OER capacity of HCNCs in the NIR light region. Additionally, it is worth mentioning that CuPc as a photosensitizer (PS) itself inevitably consumes a certain amount of O_2_ in the PDT process, which suggests that the O_2_ production rate of HCNCs is faster than the PDT O_2_ consumption rate of CuPc. The ROS generation efficiency of HCNCs under a 660 nm laser was further investigated using 1,3-diphenylisobenzofuran (DPBF) as a UV-vis sensor (Fig. 2E and S21A and B†). Under normoxic conditions, the HCNs, CuPc and HCNCs exhibit an increasing ROS production tendency, indicating that the PDT effect of HCNCs is improved. In the hypoxia environment, the ROS generated by HCNs and CuPc are extremely inhibited due to the low concentration of O_2_, while the HCNCs still maintain a comparative ROS level under normoxic conditions. The light irradiation time was optimized to be 10 min (Fig. S21C†). These results demonstrate that HCNC-driven photosynthesis-mimicking nOER-tPDT could overcome the PDT inhibition caused by hypoxic TME.

Interestingly, we also discovered enhanced POD properties of the developed HCNCs, which are able to orient CDT. In sharp contrast to single HCNs, CuPc and the mixture of the two components, the HCNCs rapidly catalyze 3,3′,5,5′-tetramethylbenzidine (TMB) oxidation in the presence of H_2_O_2_ (Fig. 2G), verifying its nanozyme properties. Also, the HCNCs could rapidly catalyze 2,2′-azinobis [3-ethylbenzothiazoline-6-sulfonic acid]-diammonium salt (ABTS) and *o*-phenylenediamine (OPD) conversion to a green and yellow product with a higher catalytic velocity, respectively, which confirms that the HCNC composite is a universal POD nanozyme (Fig. S22 and S23†). Electron spin resonance (ESR) spectroscopy analysis validates the highest ROS generation efficiency of HCNCs in the presence of H_2_O_2_ (Fig. S24†). The enzyme-like catalytic performance of HCNCs is still satisfactory at a relatively low pH value, H_2_O_2_ and particle concentrations, which is suitable for the actual TME (Fig. S25 and 26†). The Michaelis–Menten constant (*K*_m_) of the HCNCs (Fig. 2H) was determined to be 25 mM for H_2_O_2_ with a *V*_max_ of 1.88 × 10^−7^ M s^−1^, indicating a comparable affinity for H_2_O_2_ and a higher velocity compared with natural HRP and recently reported nanozymes.^38,39^ Thus, the developed HCNCs could also serve as a TME-responsible nanozyme to produce ROS for specific tumor clearance. Moreover, the TEM image, *in vitro* tPDT and POD tests demonstrate the HAase-mediated catalytic interface exposure and catalytic performance recovery of HCNCHs (Fig. S27†), which is vital for further bioapplications.

### Mechanism for enhanced CM-SERS and photosynthesis-mimicking activities of HCNCs

**Fig. 3:**
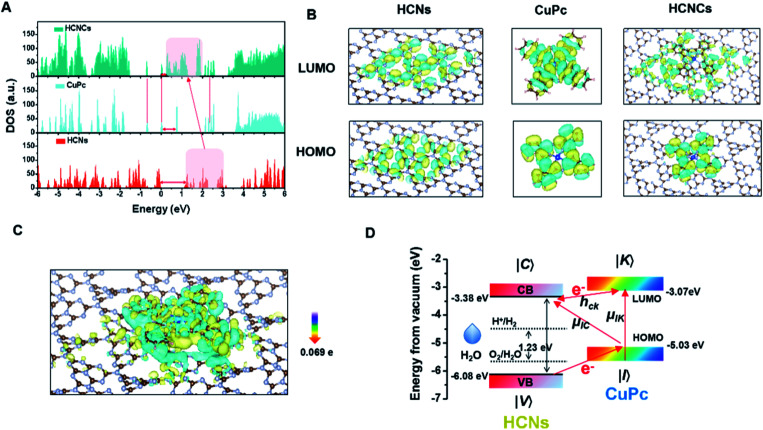
(A) DOS calculation and (B) molecular orbital densities at the HOMO and LUMO levels of the HCNs, CuPc and HCNCs. (C) The difference of charge distribution on HCNCs. (D) Schematic energy-level diagrams of CuPc on HCNs with respect to the vacuum level.

To understand these fascinating photo-responsive interfacial activities of the HCNCs, the PICT process of HCN, CuPc and HCNC systems were systematically investigated by density functional theory (DFT) calculations using the Vienna *Ab-initio* Software Package (VASP) (Fig. S28†). As shown in Fig. 3A, the HCNCs exhibit the smallest band gap and highest electronic density of states (DOS) intensity compared with HCNs and CuPc, in which the DOS of HCNs is overlapped with that of CuPc and results in a re-alignment of the total DOS distribution. This indicates strong energy level coupling between the closely contacted HCNs and CuPc in the HCNCs, which allows more possible thermodynamic PICT excitations at a low energy level and therefore effectively enhances the Raman signal, nOER and tPDT simultaneously. Meanwhile, DFT calculations of charge distributions show that the electron clouds in the HCNC system undergo a distinct migration route from CuPc to HCNs in the transition from the highest occupied molecular orbital (HOMO) to the lowest unoccupied molecular orbital (LUMO) (Fig. 3B). This indicates the successful formation of the surface CT complex with strongly increased molecular polarizability, in which the polarization tensor α is relevant to the CT resonances and molecular resonance.^40^ Furthermore, quantitative charge difference calculation in Fig. 3C shows an obvious interfacial electron migration from CuPc to HCNs, with a CT integral of 0.069 e/molecule.

Given all this, the PICT-enhanced CM-SERS and photosynthesis-mimicking mechanism was proposed as illustrated in Fig. 3D. In the CM-driven Raman system, the Raman reporter CuPc is first resonantly excited under 633 nm (1.96 eV), in which the molecular transitions (*μ*_IK_) contribute largely to the total Raman enhancement. Meanwhile, the LUMO (−3.07 eV)^41^ of CuPc and CB (−3.38 eV)^27^ of HCNs approach each other and are coupled, which allows efficient vibronic coupling for PICT transitions. The PICT transition transfers charge from the CuPc ground states (|I〉) to the HCN CB states (|C〉) *via* the transition moment *μ*_IC_, borrowing intensity from molecular transitions through the Herzberg–Teller coupling term (*h*_CK_).^42^ In this way, the electron transition and molecule polarizability are significantly augmented, bringing about a remarkable SERS enhancement. On the other hand, for the photosynthesis-mimicking process, the resonantly excited electron on the LUMO of CuPc is injected into the CB of HCNs because of the relatively lower energy level of the CB, which enhances the reducing activity of the HCNs under NIR excitation. Meanwhile, the excitation of electrons from the HOMO (−5.03 eV) to the LUMO leaves holes in the HOMO of CuPc. The close energy levels (energy gap of 1.05 eV) of the VB (−6.08 eV) of HCNs and the HOMO of CuPc make it easy for the electron in the VB of HCNs to migrate to the HOMO level of CuPc to recombine with holes of CuPc and leave holes on the VB of HCNs under 660 nm (1.88 eV) excitation, by which the electron–hole pairs are generated. Thus, the photosynthesis-mimicking OER with the redox potentials located in the HCN band gap could be initiated under NIR excitation, which provides sufficient O_2_ for the tandem PDT. Meanwhile, this process greatly enhances the number of electrons on the HOMO of CuPc and CB of HCNs, which means that the charge separation results in a higher electron supply for the above PICT transition. Hence, through the interfacial PICT line of the HCNCs, the heterogeneous composite could simultaneously achieve remarkable SERS and NIR-photocatalysis enhancement.

Furthermore, the POD reaction mechanism on the HCNCs was studied by DFT simulation (Fig. S29†). The energy barrier from the rate-determining step of HCNCs is smaller than that of HCNs and CuPc, demonstrating that the homolytic dissociation of the adsorbed H_2_O_2_ entity is more favorable on HCNCs, which thus suggests a stronger POD-like activity.

### *In vitro* Raman imaging, intracellular O_2_ and ROS evolution, and synergistic therapeutic evaluation of HCNCHs

**Fig. 4:**
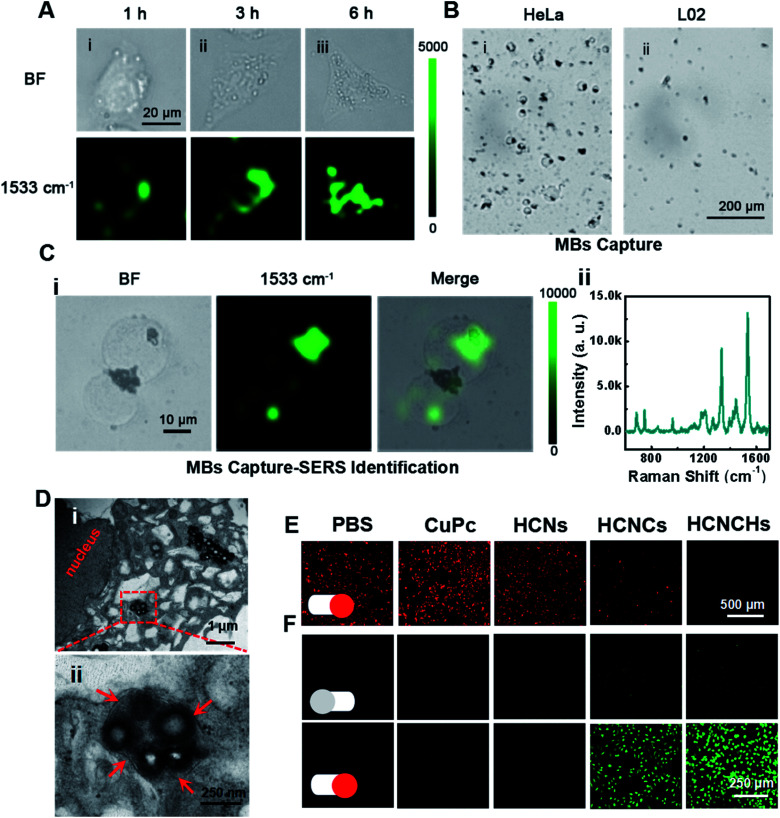
(A) Bright-field microscopy images and SERS images of HeLa cells incubated with the HCNCHs for 1, 3, and 6 h, respectively. (B) Bright-field microscopy image of the MBs@AS1411-captured (i) HeLa and (ii) L02 cells. (C) (i) SERS mapping images and (ii) SERS spectrum of the MBs@AS1411 captured HeLa cell-HCNCH complex. (D) TEM images of the subcellular distribution of the HCNCHs after incubation with HeLa cells for 6 h. (E) CLSM imaging of the intracellular O_2_ level in hypoxia-incubated HeLa cells treated with PBS, CuPc, HCNs, HCNCs and HCNCHs under light irradiation (660 nm, 0.282 W cm^−2^). (F) CLSM imaging of the intracellular ROS level in HeLa cells treated with PBS, CuPc, HCNs, HCNCs and HCNCHs in the absence or presence of light irradiation (660 nm, 0.282 W cm^−2^).

Furthermore, the intracellular behaviors of the HCNCHs were evaluated by SERS imaging. As displayed in Fig. 4A, benefiting from the HA-CD44 receptor interaction facilitated recognition and endocytosis,^43^ the SERS imaging of the HeLa cell profile shows a gradually brightened tendency from the cell membrane region into the cell with prolonging incubation time, and reaches an integrated cell image in 6 h. We further constructed a capture-identification SERS diagnostic platform for fast *in vitro* detection of circulating tumor cells (CTCs) (illustrated in Scheme S1†). As displayed in the bright field in Fig. 4B, using AS1411 aptamer^44^-modified magnetic beads (MBs@AS1411) as the capturing hook, HeLa CTCs are rapidly extracted through specific recognition between the aptamer and the overexpressed nucleolar protein on the cell membrane. However, no L02 normal cell is caught, indicating the specificity of MBs@AS1411 in achieving the separation of tumor cells and normal cells. Then, by introducing the HCNCHs into the magnetic separation system, a distinct SERS imaging profile was achieved with a robust SERS signal readout at the single-cell level (Fig. 4C). These results reveal the efficiency, specificity and sensitivity of this HCNCH-based capture-identification SERS diagnostic platform, which holds prominent potential in cancer early diagnosis and SERS-guided therapy evaluation. Meanwhile, the confocal laser scanning microscopy (CLSM) co-localization staining result validates that the HCNCHs are effectively entrapped into the lysosomes and endosomes (Fig. S30–S32†). Also, the subcellular particle location was confirmed by bio-TEM. As expected in Fig. 4D, masses of HCNCHs are distributed into the cytoplasm and encapsulated by the cytosolic vesicles of HeLa cells after 6 h incubation, which is consistent with the SERS and CLSM results.

Furthermore, we investigated the intracellular hypoxic TME modulation, tPDT and TME-responsive CDT effects of the HCNCH composite for multi-mode therapy. Using [Ru(dpp)_3_]Cl_2_ (RDPP) as an O_2_ probe,^45^ a HCNCH-incubated HeLa cell under 660 nm irradiation and hypoxic conditions shows faded red FL, indicating that the HCNCs derive the water-splitting reaction inside the cell under NIR excitation, and meanwhile the HA layer enhances the internalization of the HCNCHs into the tumor cell to enrich the nanocatalysts for more effective hypoxia relief (Fig. 4E). Furthermore, 2′,7′-dichlorofluorescein diacetate (DCFH-DA) was used as an intracellular ROS sensor to evaluate the combined tPDT and CDT in HeLa cells. As displayed in Fig. 4F, the strongest green FL is detected in HCNCH incubated HeLa cells, which suggests that the CDT, nOER-enhanced tPDT effect and the HA-elevated cellular endocytosis great enhance the ROS generation. The synergistic therapeutic efficiency of the tPDT and CDT of the HCNCHs was systematically investigated using MTT assay. As shown in Fig. S33A,† a much higher inhibition of cell proliferation was observed in the HCNCH-treated HeLa cells under the same drug treatments, revealing an accelerated nOER-tPDT process accompanied by simultaneous CDT. Meanwhile, the dose-dependent viability inhibition data in Fig. S33B† show a much higher increasing inhibition of cell proliferation in the HCNCH-treated HeLa cells with a cell viability of less than 20%, demonstrating the superiority of the combination of CDT and nOER-tPDT. Also, the HCNCH-treated L02 cells exhibit more than 90% cell viability due to the negligible affinity, which thus could effectively reduce the toxic side effects on normal tissue (Fig. S34†). Furthermore, CLSM analysis results using calcein-AM and propidium iodide (PI) to co-stain dead/live cells are consistent with the aforementioned cell viability results, confirming that the combined therapeutic formulation is most effective in killing cancer cells (Fig. S35†).

### *In vivo* Raman monitoring of drug delivery and antitumor therapeutic effect evaluation

**Fig. 5:**
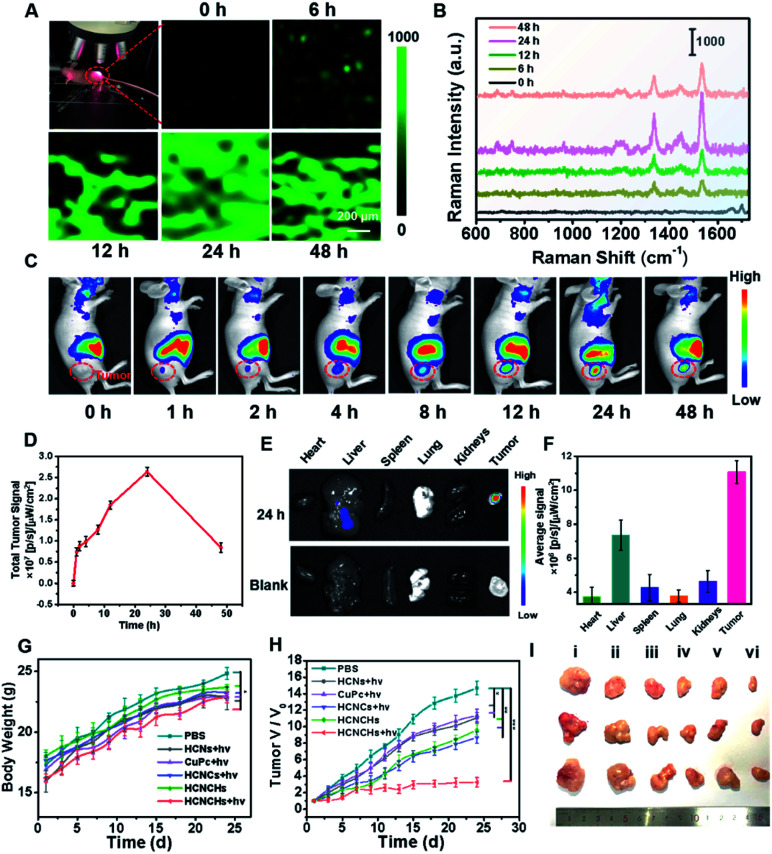
(A) *In vivo* Raman imaging system with a laser beam focusing on the tumor and the SERS image of the tumor at 1533 cm^−1^ at different post-injection times of HCNCHs using a 5× working objective lens on the tumor, under 633 nm laser excitation, 17 mW laser power, and an exposure time of 1 s. (B) Typical SERS spectra acquired from the tumor region at different post-injection times of HCNCHs. (C) *In vivo* FL imaging of Cy5.5-labeled HCNCHs at different post-injection times and (D) the corresponding FL intensities in the tumor region. (E) *In vivo* FL imaging and (F) signal intensities of the major organs and tumor at 24 h postinjection. (G) Body weight changes, (H) tumor volume changes, and (I) tumor images of mice after treatments of PBS, CuPc + light, HCNs + light, HCNCs + light, HCNCHs and HCNCHs + light.

Encouraged by the excellent *in vitro* theranostic performance of the HCNCH nanoplatform, we further systematically investigated its *in vivo* behaviors. We first traced its dynamic distribution by *in vivo* SERS imaging. HeLa tumor bearing nude mice were anesthetized after injection and a 633 nm Raman laser was focused on the tumor site for SERS measurement, using the edge mode with an exposure time of 1 s per pixel. As shown in Fig. 5A and B, the Raman signal at 1533 cm^−1^ of HCNCHs is detectable after 6 h injection, which then increases with prolonging time and reaches the brightest level at 24 h postinjection, demonstrating the effective retention of the HCNCHs at the tumor site. Subsequently, the SERS signal declines to a relative low level during the following 24 h, indicating the gradual metabolization of the nanodrug. Meanwhile, *in vivo* FL imaging of Cy5.5-labeled HCNCHs (Fig. S36†) exhibits a similar “volcano” tendency with the most intense intensity at 24 h, which therefore is chosen as the implementing time point for PDT light irradiation (Fig. 5C and D). Also, the bio-distribution of the HCNCHs in the major organs and tumor demonstrates that the HCNCH-received mouse presents a bright FL signal in the tumor with some inevitable accumulation in the liver, confirming the highly targeting ability of the HCNCHs (Fig. 5E and F). Accordingly, the above *in vivo* Raman and FL imaging results validate the specificity of the as-proposed HCNCHs delivering to target tumor tissue, which thus provides a clear imaging guidance for further precise cancer therapy. Furthermore, the pharmacokinetic profile of the HCNCHs shows the liver clearance pathway, which cannot induce a significant immune response at the dose used in this work (Fig. S37†).

The synergistic anti-cancer efficiency of HCNCHs was then examined in HeLa tumor-bearing nude mice. The tumor-bearing nude mice were randomly divided into 6 groups (*n* = 3): PBS, CuPc + light, HCNs + light, HCNCs + light, HCNCHs and HCNCHs + light. As the therapeutic schedule shown in Scheme S2,† the intravenous injection was administrated on the 1st, 7th and 16th days and the irradiation groups were treated with a 660 nm laser at 24 h postinjection. The mice weight of the various groups shows a similar increasing tendency during the 24 day treatment, evidencing the *in vivo* biocompatibility of the series of nanodrugs (Fig. 5G). The tumor growth of the mice after various treatments was monitored (Fig. 5H and I and S38†). Remarkably, the HCNCHs + light group exhibits the highest tumor inhibition rate of 77.7% because of the excellent synergistic antitumor therapeutic efficacy of the HA specific targeting-assisted nOER-tPDT/CDT synergistic therapy. In addition, histology and immunohistochemical analysis by hematoxylin-eosin staining (H&E) and terminal deoxynu-cleotidyl transferase dUTP nick-end labeling (TUNEL) staining assay verify the supreme tumor inhibition effect of the combined therapy strategy (Fig. S39†). Furthermore, the potential systemic toxicity of the HCNCHs + light treatment was investigated. Histological analysis of the major organs based on H&E staining in Fig. S40† reveals that the nOER-tPDT and CDT combinational therapy induces little histological alteration. The according ICP-AES analysis of the Cu accumulation in major organs after 24 day therapeutic treatment of HCNCHs reveals a relatively complete metabolization of the HCNCHs (Fig. S37†). Additionally, normal serum biochemistry and hematology analysis demonstrates negligible hepatic or renal toxicity and remarkable biocompatibility of the HA-targeted therapeutic strategy (Fig. S41†).

## Conclusions

In summary, we have successfully developed a biomimetic HCNCH nanoplatform integrating chemical enhanced SERS and photosynthesis-mimicking properties for theranostic applications. HCNs adhered with a uniform CuPc molecule film mimics a photo-pigment modified photosynthetic membrane for fast charge separation and electron transport. Thus, the thylakoid-inspired nanoplatform plays two key roles as a light-harvesting nanocomposite and photosynthetic-mimicking nanoscaffold that enhance both CM-SERS and photoredox catalysis. The application of the heterostructure has been demonstrated in cancer theranostics. Fast SERS capture-detection-imaging platforms at the single-cell level and *in vivo* SERS imaging were achieved for CTC diagnosis and real-time monitoring of drug delivery. Photosynthesis-mimicking nOER-photodynamic catalysis and POD performances were applied in hypoxic TME-modulating/responding photo-chemodynamic therapy of cervical cancer with an excellent outcome. We believe that this study opens up a new avenue for biomimetic CM-SERS nanoplatform design and warrants widespread biomedical applications for diagnosis, imaging, and disease treatment.

## Data availability

All experimental and characterization data, as well as DFT calculation data are available in the ESI.†

## Author contributions

Yu Su, Baozhen Yuan and Yaowen Jiang contributed equally to this work. Yu Su conceived the idea and designed the experiments. Yu Su performed the material performance test and analysed the data. Baozhen Yuan and Yaowen Jiang performed the *in vitro* and *in vivo* theranostic experiments. Yu Su analyzed the DFT simulations. Yu Su, Baozhen Yuan and Yaowen Jiang created figures. Yu Su wrote the paper. Jun-Jie Zhu and Li-Ping Jiang discussed the results and commented on the paper. Ping Wu and Xiaolin Huang supervised the project and involved in manuscript preparation.

## Conflicts of interest

There are no conflicts to declare.

## Supplementary Material

SC-013-D2SC01534J-s001
