# Supplementary material for: A bioinspired hollow g-C3N4–CuPc heterostructure with remarkable SERS enhancement and photosynthesis-mimicking properties for theranostic applications
Source: Chem Sci. 2022 May 12;13(22):6573–82. doi: 10.1039/d2sc01534j (PMC9172571; doi:10.1039/d2sc01534j)
Supplement: SC-013-D2SC01534J-s001 [file SC-013-D2SC01534J-s001.pdf]

Electronic Supplementary Material (ESI) for Chemical Science.
This journal is © The Royal Society of Chemistry 2022

# Supporting Information

# Bioinspired hollow g-$C_3N_4$-CuPc heterostructure with remarkable SERS enhancement and photosynthesis-mimicking property for theranostic applications

*Yu Su[1,2], Baozhen Yuan[1], Yaowen Jiang[1], Ping Wu[3], Xiaolin Huang[2], Jun-Jie Zhu[1,4]*and Li-Ping Jiang[1]**

[1]State Key Laboratory of Analytical Chemistry for Life Science, School of Chemistry and Chemical Engineering, Nanjing University, Nanjing, Jiangsu 210023, China

[2]State Key Laboratory of Food Science and Technology, School of Food Science and Technology, Nanchang University, Nanchang 330047, China

[3]Jiangsu Key Laboratory of New Power Batteries, College of Chemistry & Materials Science, Nanjing Normal University, Nanjing, Jiangsu 210097, China

[4]Shenzhen Research Institute of Nanjing University, Shenzhen 518000, China

*To whom correspondence should address at jjzhu@nju.edu.cn; jianglp@nju.edu.cn.

**Table of Contents**

**Experimental Section** ......................................................................................................3

**Additional Results and Discussion**......................................................................................16

1. Characterization of the HCNCs..........................................................................................16

2. SERS Study of HCNCs......................................................................................................20

3. Photosynthetic-Mimicking nOER-tPDT Performance of the HCNCs................................28

4. Peroxidase-Mimicking Characterization of the HCNCs....................................................29

5. DFT Calculations................................................................................................................32

6. Characterization of the HCNCHs and the Intracellular Study............................................34

7. *In Vivo* Assay........................................................................................................................38

**References**.............................................................................................................................43

**Experimental Section.**

**Chemicals and Materials.**

n-Octadecyltrimethoxysilane ($C_{18}$TMOS), ammonium hydrogen fluoride ($NH_4HF_2$) were purchased from Macklin Biochemical Co., Ltd. Cyanamide, copper (II) phthalocyanine (CuPc), hyaluronidase, 2',7'-dichlorfluorescin diacetate (DCFH-DA), $[Ru(dpp)_3]Cl_2$ (RDPP), 5,5-dimethyl-1-pyrroline-N-oxide (DMPO) were purchased from Sigma Aldrich (St. Louis, MO). 1,3-Diphenylisobenzofuran (DPBF), 3,3',5,5'-tetramethylbenzidine (TMB), [3-ethylbenzothiazoline-6-sulfonic acid]-diammonium salt (ABTS), o-phenylenediamine (OPD), streptavidin, hyaluronic acid (HA) were purchased from Aladdin Reagent Co., Ltd. Tris(hydroxymethyl)aminomethane (Tris), hydrogen peroxide ($H_2O_2$), dimethyl sulfoxide (DMSO), glutaric dialdehyde were purchased from Sinopharm Chemical Reagent Co., Ltd. (China). Calcein AM and propidium iodide (PI) were bought from Dojindo Molecular Technologies, Inc. 3-(4,5-dimethyl-2-thiazolyl)-2,5-diphenyl-2-H-tetrazolium bromide (MTT) and 4',6-diamidino-2-phenylindole (DAPI), LysoTracker Red, Dulbecco's modified Eagle's medium (DMEM), phosphate buffer saline (PBS, 10 mM, pH 7.4) were obtained from Keygen Biotechnology Co., Ltd. (Nanjing, China). The oligonucleotides as listed in Supplementary Table 1 were synthesized and purified by Sangon Biotechnology Co. Ltd. (Shanghai, China). TE buffer containing 10 mM Tris-HCl, 1 mM EDTA, 12.5 mM $MgCl_2$, 20 mM $K^+$ (pH 7.4) was used as the DNA buffer. 0.2 M HAc/NaAc (pH 5.5) buffer was used as the TMB oxidation buffer. All the chemicals were of analytical grade and used as received without further purification.

**Apparatus and Characterizations.**

High resolution transmission electron microscopy (HRTEM) images and EDX images were taken using a JEM-2800 with an accelerating voltage of 200 kV. The high-angle annular dark-

field scanning transmission electron microscopy (HAADF-STEM) image was taken using a Titan G2 60-300. UV-vis spectra were recorded on a UV-3600 spectrophotometer (Shimadzu, Kyoto, Japan). Fourier transform infrared (FT-IR) spectra were measured by a Nicolet 6700 spectrophotometer (Nicolet Co., USA). Small angle powder X-ray diffraction (XRD) measurement was conducted on a Thermo ARL SCINTAG X'TRA diffractometer using a Cu-Kα radiation (λ = 0.15405 nm). PL spectra were measured with an F-7000 spectrophotometer (Hitachi, Japan). PL lifetime was recorded by an FLS980 PL spectrometer (Edinburgh, England). X-ray photoelectron spectroscopy was analyzed by an ESCALAB250Xi spectrometer (Thermo Fisher Scientific Co., USA). The Brunauer-Emmett-Teller (BET) specific surface area was calculated according to adsorption data in low pressure and the corresponding pore diameter was estimated using the Barrett-Joyner-Halenda (BJH) method. Dynamic light scattering (DLS) experiments were performed at 25 °C using a Brookhaven BI-200SM instrument, equipped with a He-Ne laser (632.8 nm) at a fixed scattering angle of 90º. Zeta-potentials of colloidal NPs were measured at room temperature using ZETASIZER nanoseries (Nano-ZS, Malvern). Atomic absorption spectroscopy was measured by analytik jena novAA350/ZEEnit650p spectrometer (Analytik Jena AG, Germany). Electron spin resonance (ESR) spectra were measured by Bruker EMX plus spectrometer. Thermogravimetric curves were measured using ASAP2020 thermoanalyser (NETZSCH, Germany). Fluorescence (FL) imaging was performed using confocal laser scanning microscopy (CLSM) on a Leica TCS SP8 confocal microscope (Leica Microsystems Inc., Exton, PA). SERS spectra and images were obtained using a Renishaw inVia-Reflex Raman microscope system (Renishaw, U. K.). A Helium-neon laser at 633 nm was used for excitation, and spectra were acquired using a 20× working objective lens on a sample at

laser power of 1.7 mW and exposure time of 10 s. The *in vivo* FL images were monitored by a PE IVIS Lumina XR III (U. S. A.).

**Synthesis of the HCN nanospheres.**

A dense monodisperse silica core covered with a thin mesoporous silica shell was used as a template to synthesize the hollow carbon nitride spheres.[1] In detail, 1 g of mesoporous $SiO_2$ template and 5 mL of cyanamide were mixed for 3 h in a flask connected to a vacuum line before subjecting to sonication at 60 °C for 8 h. Afterwards, the mixture was stirred at 60 °C overnight. The resultant mixture was centrifuged and dried in air to obtain a white solid that was transferred to a crucible and heated to 550 °C under flowing $N_2$ for 4 h with a ramp rate of 4.4 °C $min^{-1}$. The obtained yellow g-$C_3N_4$/$SiO_2$ hybrids were treated with 4 M $NH_4HF_2$ for overnight to remove the silica template. The powders were then centrifuged and washed three times with distilled water and once with ethanol. The final yellow HCNs powders were obtained by drying at 60 °C in a vacuum oven for 10 h.

**Synthesis of the HCNCHs.** The HCNs-CuPc conjugate (HCNCs) was prepared by one-pot solvothermal self-assembly. In detail, 5 mg of HCNs powder was dissolved in 10 mL of 250 μM CuPc solution and the mixture were sonicated for 1 h. Then, the mixture was transferred into a Teflon-lined autoclave, sealed and heated at 120 °C for 12 h. After the system was cooled down to room temperature, the blue product was washed with distilled water by centrifugation at 8000 rpm for 10 min to remove excess CuPc molecule for 3 times and redispersed in 5 mL of distilled water. Then, HA-modified HCNCs (HCNCHs) was prepared for better targeting ability and biocompatibility. In detail, 250 μL 1 mg $mL^{-1}$ HA solution was added into 500 μL 1 mg $mL^{-1}$ HCNC and stirred overnight at room temperature, followed by centrifugation at 8000 rpm for 10

min and washed with water for 3 times. The obtained HCNCHs was redispersed in 500 μL PBS and stored at 4 °C for further use.

**Preparation of FAM/Cy5.5-labeled HCNCHs.**

FAM and Cy5.5-labeled HCNCHs were prepared for *in vitro* and *in vivo* fluorescence imaging, respectively. Briefly, 10 μL of 100 μM FAM-$C_{20}$ or Cy5.5-$C_{20}$ was added in 250 μL of 1 mg $mL^{-1}$ HCNCs and stirred overnight at room temperature for the interaction between HCNCs and DNA, followed by adding 125 μL of 1 mg $mL^{-1}$ HA solution and stirred for another 12 h at room temperature. The suspension was then centrifugated at 8000 rpm for 10 min and washed with PBS for 3 times. The obtained fluorochrome-labeled HCNCHs was redispersed in 500 μL PBS with a final concentration of 500 μg $mL^{-1}$ and stored at 4 °C for further use.

**Preparation of the Apt-MBs**.

50 μL of 25 % glutaric dialdehyde was added into 500 μL of 1 mg $mL^{-1}$ aminated magnetic beads (MBs) and stirred for 1 h at room temperature. The MBs were separated and washed using a magnet, and redispersed in 500 μL DI water. Then, 100 μL of 1 mg $mL^{-1}$ streptavidin was added into the MBs suspension with continuous stirring for 12 h, and the obtained streptavidin-modified MBs were washed with DI water using a magnet for 3 times and redispersed in 500 μL PBS with a concentration of 1 mg $mL^{-1}$ for further use. For aptamer modification, 10 μL of 100 μM biotin-AS1411 was mixed with 200 μL of the streptavidin-modified MBs and stirred for 6 h at room temperature. The MBs@AS1411 were washed with PBS using a magnet for 3 times and redispersed in 50 μL PBS and stored at 4 °C for further use.

***In Vitro* $O_2$ Evolution Analysis.**

10 mL 100 μg $mL^{-1}$ of HCNCs suspension was added in a 10 mL three-necked flask with $N_2$ bubbling for 30 min to remove the dissolved oxygen. Then, the suspension was exposed to continuous laser irradiation (660 nm, 0.282 W $cm^{-2}$) and a AR8010 portable dissolved oxygen meter (SMART SENSOR Instrument Factory) was utilized to record the time-dependent oxygen generation.

***In Vitro* ROS Detection.**

The extracellular ROS was detected using DPBF as chemical probe. Typically, 2 μL of 2 mg $mL^{-1}$ DPBF solution was added in 20 μL of 100 μg $mL^{-1}$ normoxia and hypoxia HCNs, HCNCs suspension and corresponding CuPc solution. The mixture was irradiated with a 660 nm laser (0.282 W $cm^{-2}$) for 10 min, and the absorbance intensity of DPBF at 420 nm was recorded every 2 min.

**Peroxidase-like Activity and Kinetics Assay.**

All kinetic measurements were monitored in a time-scan mode at 653 nm through a UV-3600 spectrophotometer (Shimadzu, Kyoto, Japan). The steady-state kinetic assay was performed at room temperature. The final working concentrations were 1 mM and 10 mM for TMB and $H_2O_2$, respectively. The reaction time was 30 min, and the buffer solution was pH 5.5 HAc/NaAc buffer (0.2 M). The Michaelis−Menten constant was calculated according to $v=V_{max}\times[S]/(K_m+[S])$, where $V_{max}$ represents the maximal reaction velocity, [S] is the concentration of the substrate, and $K_m$ is the Michaelis constant.

**Calculation of the SERS Enhancement Factor (EF).**

The enhancement factor EF was calculated according to the following equation:

$$EF = \frac{I_{SERS} * N_{bulk}}{I_{bulk} * N_{ads}} \quad (1)$$

$N_{ads}$ and $N_{bulk}$ represent the number of CuPc molecules in the SERS sample and the normal Raman sample, respectively. $I_{SERS}$ and $I_{bulk}$ are the same vibration peak of CuPc molecule on HCNCs and the normal Raman spectrum from solid sample, respectively. In the experiment, 100 μL of aqueous CuPc solution (0.1 M) was dried onto the Si wafer (0.5 × 0.5 $cm^2$ ) and $N_{bulk}$ can be estimated as:

$$N_{bulk} = 100\ \mu L \times 10^{-1}\ mol\ L^{-1} \times 6.02 \times 10^{23}\ mol^{-1} \times 2.93\ \mu m^2 / 0.25\ cm^2 \quad (2)$$

where d is the diameter of the laser spot, d = 1.22 $\lambda/N_A$, λ is incident wavelength 633 nm, the numerical aperture of the objective lens $N_A$ = 0.4, thereby, laser spot size ($\pi (d/2)^2$) is about 2.93 $\mu m^2$. $N_{bulk}$ was estimated to $7.06 \times 10^{11}$.

$N_{ads}$ is determined by laser spot illuminating on the sample and density of CuPc molecule adsorbed on the surface of HCNs. The CuPc loading amount of HCNCs was quantified to be 0.11 μmol $mg^{-1}$. Thus, the $N_{ads}$ can be estimated to be $7.76 \times 10^7$ according to the following equation:

$$N_{ads} = 100\ \mu L \times 100\ \mu g\ mL^{-1} \times 0.11\ \mu mol\ mg^{-1} \times 6.02 \times 10^{23}\ mol^{-1} \times 2.93\ \mu m^2 / 0.25\ cm^2 \quad (3)$$

$I_{SERS}$ and $I_{bulk}$ were obtained on the peak intensity of CuPc molecule at 1533 $cm^{-1}$ in SERS spectrum and normal Raman spectrum, where $I_{SERS}$ = 176255 and $I_{bulk}$ = 8724 (**Figure S18**). Substituting these values of the above variables into equation (1), EF could be calculated to be around $1.84 \times 10^5$.

**Computational Methods.**

All the calculations were performed within the framework of the density functional theory (DFT) as implemented in the Vienna *Ab-initio* Software Package (VASP 5.3.5) code within the Perdew-Burke-Ernzerhof (PBE) generalized gradient approximation and the projected augmented wave (PAW) method.[2-6] The cutoff energy for the plane-wave basis set was set to 400 eV. The Brillouin zone of the surface unit cell was sampled by Monkhorst-Pack (MP) grids, with a k-point mesh for CuPc and $C_3N_4$-based structure optimizations.[6] The CuPc molecule was determined by Γ Monkhorst-Pack grid and the $C_3N_4$-based surfaces were determined by 2 × 2 × 1 Monkhorst−Pack grid. The convergence criterion for the electronic self-consistent iteration and force was set to $10^{-5}$ eV and 0.01 eV/Å, respectively.

The free energies of adsorbates and transition states at temperature T(*S(T)*) were estimated according to the harmonic approximation, and the entropy is evaluated using the following equation:

$$S(T) = k_{\mathrm{B}} \sum_{i}^{\text{harm DOF}} \left[ \frac{\varepsilon_i}{k_{\mathrm{B}}T(\mathrm{e}^{\varepsilon_i/k_{\mathrm{B}}T}\text{-}1)} \text{-}\ln(1\text{-}\mathrm{e}^{\text{-}\varepsilon_i/k_{\mathrm{B}}T}) \right] \quad (4)$$

where $k_B$ is Boltzmann's constant and DOF is the number of harmonic energies ($\varepsilon_i$) used in the summation denoted as the degree of freedom, which is generally 3N, where N is the number of atoms in the adsorbate or transition state. Meanwhile, the free energies of gas phase species ($G_g(T)$) are corrected as:

$$G_{\mathrm{g}}(T) = E_{elec} + E_{ZPE} + \int C_p \mathrm{d}T - TS(T) \quad (5)$$

where $C_p$ is the gas phase heat capacity as a function of temperature derived from Shomate equations, $E_{elec}$ is electronic energy and $E_{ZPE}$ is correction for zero point energy. The corresponding parameters in the equations were obtained from National Institute of Standards and Technology.

**Cell Culture in Hypoxia and Normoxia Environments.**

Human cervical cancer cells (HeLa cells) and human normal liver cells (L02 cells) were obtained from the Cell Bank of Type Culture Collection of Chinese Academy of Sciences (Shanghai, China). These cells were respectively cultured in DMEM, supplemented with 10 % FBS and penicillin streptomycin (100 U/mL) at 37 °C in a humidified incubator containing 21 % $O_2$, 5 % $CO_2$ and 1 % $O_2$, 5 % $CO_2$ to mimic the normoxia and hypoxia environment, respectively. The cells at the end of the log phase were selected for the following experiments.

***In vitro* SERS Imaging and SERS Detection.**

*In vitro SERS Imaging*: To monitor the cellular internalization of the HCNCHs, HeLa cells were seeded on confocal dishes and incubated at 37 °C for 12 h. Then, the HeLa cells were incubated with 1 mL of the HCNCHs suspension (50 μg $mL^{-1}$) in DMEM at 37 °C for 1, 3, and 6 h. After that, the cells were washed three times with PBS to remove excess HCNCHs. The single cell-SERS images were obtained using the Renishaw inVia-Reflex Raman microscope system with 50× working objective lens, a scanning step of 3 μm, a 633 nm excitation, a 17 mW laser power, and an exposure time of 1 s.

*SERS Capture-Identification Diagnostic Platform*: For the circulating tumor cells (CTCs)-specific capture-recognition, aptamer-modified MBs were used as the capturing platform. In brief, HeLa or L02 cells were mixed with 0.1 mL of HCNCHs and 1 μL MBs@AS1411 and incubated at 37 °C for 1 h. The MBs-cell-HCNCHs complex were separated and washed by PBS

3 times using a magnet, and redispersed in 10 μL PBS. The obtained solution was dropped on a glass slide and covered by a coverslip for bright field imaging and SERS detection.

**Intracellular Endocytosis Analysis.**

HeLa or L02 cells were seeded on confocal dishes and incubated at 37 °C for 12 h. Then, the cells were incubated with 1 mL of the FAM-HCNCHs suspension (50 μg $mL^{-1}$) in DMEM at 37 °C for 1, 3, and 6 h. After that, the cells were washed three times with PBS to remove excess FAM-HCNCHs (Ex: 488 nm, Em: 520 nm). Then, the cells were lysosome stained with Lysotracker Red DND-99 (Ex: 575 nm, Em: 600 nm) for 30 min and nuclei stained with DAPI (Ex: UV, Em: 460 nm) for 5 min. Finally, the cells were washed with PBS for 3 times and added in fresh medium for confocal laser scanning microscopy (CLSM) observation.

**Intracellular TEM Imaging.**

HeLa cells were seeded on confocal dishes and incubated at 37 °C overnight. Then, the HeLa cells were incubated with 1 mL of the HCNCHs suspension (50 μg $mL^{-1}$) in DMEM at 37 °C for 6 h. After that, the cells were washed three times with PBS to remove excess HCNCHs. Then, the cells were harvested using a cell scraper and fixed with 2.5 % glutaraldehyde in PBS at 4 °C overnight. The cells were washed with PBS three times for 15 min. Subsequently, the fixed cells were stained with uranyl acetate, and dehydrated in a graded series of acetone (50, 70, 90 and 100 %). Then, the specimens were embedded in resin and cut into the ultrathin sections of 70-90 nm thickness. After staining on the grid with a solution containing uranyl acetate and lead citrate, the state and distribution of the HCNCHs in HeLa cells were observed by TEM.

**Intracellular $O_2$ Imaging.**

The intracellular $O_2$ were measured using a $[(Ru(dpp)_3)]Cl_2$ (RDPP) probe. HeLa cells in hypoxia incubator were incubated and treated with HCNCHs (50 μg $mL^{-1}$) at 37 °C for 6 h. Then, the medium was removed and a new medium containing RDPP (10 μM) was added and hypoxialy incubated for another 30 min. Subsequently, the cells were washed with PBS and added in PBS, followed by illuminated with a 660 nm laser (0.282 W $cm^{-2}$) for 10 min. Finally, the intracellular fluorescence was measured via CLSM (Ex: 488 nm, Em: 600-650 nm). The DMEM and PBS used here were bubbled with $N_2$ for 30 min to remove dissolved $O_2$ before hypoxia environment use.

**Intracellular ROS Imaging.**

2',7'-Dichlorfluorescin diacetate (DCFH-DA) was employed to study the production of intracellular ROS by confocal analysis. In detail, HeLa cells were seeded onto a confocal microscopy dish at a density of $10^4$ cells per well and allowed to attach overnight. CuPc, HCNs, HCNCs, HCNCHs at a specific concentration were added and incubated with the cells for 6 h. For PDT, the cells were exposed to a 660 nm laser (0.282 W $cm^{-2}$) for 5 min. Then the cells were rinsed with PBS twice and stained with DCFH-DA (10 μM in serum-free medium) under 37 °C for 20 min. Finally, the treated cells were rinsed with PBS and imaged using CLSM (Ex: 488 nm, Em: 525 nm).

***In Vitro* Cytotoxicity.**

The cytotoxicity of NPs to HeLa cells and L02 cells was assessed using a 3-(4,5-dimethylthiazol-2yl)-2, 5-diphenyltetrazolium bromide (MTT) assay. In short, the HeLa cells and L02 cells (100 μL, $10^5$ cells per mL) were seeded into a 96-well cell-culture plate, followed by the incubation of CuPc, HCNs, HCNCs and HCNCHs at a specific concentration for 6 h in an

incubator (21 % $O_2$, 5 % $CO_2$, 37 °C). For PDT, the cells were exposed to a 660 nm laser (0.282 W $cm^{-2}$) for 5 min. Then, 100 μL of MTT (0.5 mg $mL^{-1}$) was added to each well and incubated for 4 h. After removing the supernatant, 100 μL DMSO was added to each well. Then the optical density (OD) was read at a wavelength of 490 nm. Relative cell viability was expressed as follows: $\% = ([OD]_{test}/[OD]_{control})\times 100$.

**Live/Dead Cell Staining.**

HeLa cells were seeded in a six-well plate at a density of $10^5$ cells per well and incubated overnight. CuPc, HCNs, HCNCs, HCNCHs were added and incubated with the cells for 6 h, and then the cells were irradiated by 660 nm laser for 5 min and rinsed with PBS twice. After detachment with trypsin and washing with PBS twice, the cells were costained by calcein AM (Ex: 490 nm, Em: 515 nm) and PI (Ex: 490 nm, Em: 617 nm) for 15 min. The FL images were captured using CLSM.

***In Vivo* SERS Imaging.**

All animal studies were performed in accordance with the Jiangsu Animal Care and Use Committee (SYXK(Su) 2017-0015). Murine tumor xenograft models were generated by subcutaneously inoculating 1 × $10^6$ HeLa cells (in 150 μL PBS) in female BALB/c athymic nude mice. When the tumor volume reached 100 $mm^3$, the mice were intravenously injected with 100 μL 630 μg $mL^{-1}$ HCNCHs (~3 mg $kg^{-1}$). Then, the mice were anesthetized at different time point and the Raman imaging was performed using a 5× working objective lens on the tumor under a 633 nm laser excitation, a 17 mW laser power, and an exposure time of 1 s using the edge scanning mode.

***In Vivo* Fluorescence Imaging.**

When the volumes of HeLa tumors reached 100 $mm^3$, the nude mice were injected with 100 μL of 630 μg $mL^{-1}$ Cy5.5-labeled HCNCHs (~3 mg $kg^{-1}$) via the tail vein. The real-time fluorescence images (Ex: 660 nm and Em: 710 nm) were carried out on a Bruker Icon *in vivo* imaging system at 0, 1, 2, 4, 8, 12, 24 and 48 h. Then the mice were euthanized and the normal organs (heart, liver, spleen, kidneys and lung) and tumor tissues were collected for *in vitro* imaging using the same system.

***In Vivo* Synergistic Antitumor Therapy.**

When the average body weight reached ~16 g, the mice were randomly divided into 6 groups (three mice per group): the control group was treated with 100 μL of PBS, while the other groups were treated with 100 μL of corresponding samples (69.3 μM CuPc + light, 630 μg $mL^{-1}$ HCNs + light, 630 μg $mL^{-1}$ HCNCs + light, 630 μg $mL^{-1}$ HCNCHs and 630 μg $mL^{-1}$ HCNCHs + light) on the 1st, 7th and 16th day. The final concentration of HCN materials was ~3 mg $kg^{-1}$, and the laser irradiation groups were irradiated with the power density of 0.282 W $cm^{-2}$ for 10 min after 24 h of injection. Meanwhile, the weight of the mice and the volume of the tumor was measured every two days. The tumor volume was calculated using the equation of $V=(\text{length}\times\text{width}^2)/2$. For histological analysis, the mice were euthanized at the end of the treatments. Subsequently, the tumors and major organs (including heart, liver, spleen, kidney, and lung) were harvested for routine staining with hematoxylin-eosin (H&E) and terminal deoxynu-cleotidyl transferase dUTP nickend labeling (TUNEL). The final images were obtained and analyzed under a microscope (Olympus BX41, Japan). Meanwhile, major organs (including heart, liver, spleen, kidneys and lung) were digested in $HNO_3$ at 120 °C overnight and the obtained yellow powders were dissolved in 3% $HNO_3$ for ICP-AES analysis. Blood samples (approximately 500 μL/sample) were collected via orbital puncture from each mouse. For serum biochemistry study,

the blood was centrifuged at 4000 rpm for 10 min and the serum was harvested. The serum biochemistry parameters include alanine aminotransferase (ALT), aspartate aminotransferase (AST), alkaline phosphatase (ALP), albumin (ALB), blood urea nitrogen (BUN), and γ-glutamyl transaminase (γ-GT). For hematology, whole blood was collected in a potassium EDTA collection tube, including white blood cell (WBC) count, red blood cell count (RBC), hemoglobin (HGB), platelet count (PLT), % of lymphocyte (LY), and % of neutrophils (NE).

**Statistical Analysis.**

Data were presented as mean ± standard deviation (SD). The statistical differences of data were evaluated by one-way analysis of variance. "*" means $p < 0.05$, which was considered to be significant. "**" means $p < 0.01$ and "***" means $p < 0.001$, which were considered to be very significant.

**Table S1.** Oligonucleotides sequences used in this study.

| Name | Sequence (5'-3') |
|---|---|
| FAM-$C_{20}$ | FAM-CCCCCCCCCCCCCCCCCCCC |
| Cy5.5-$C_{20}$ | Cy5.5-CCCCCCCCCCCCCCCCCCCC |
| biotin-AS1411 | biotin-TTTTTTTTGGTGGTGGTGGTTGTGGTGGTGGTGG |

## Additional Results and Discussion

### 1. <u>Characterization of the HCNCs.</u>

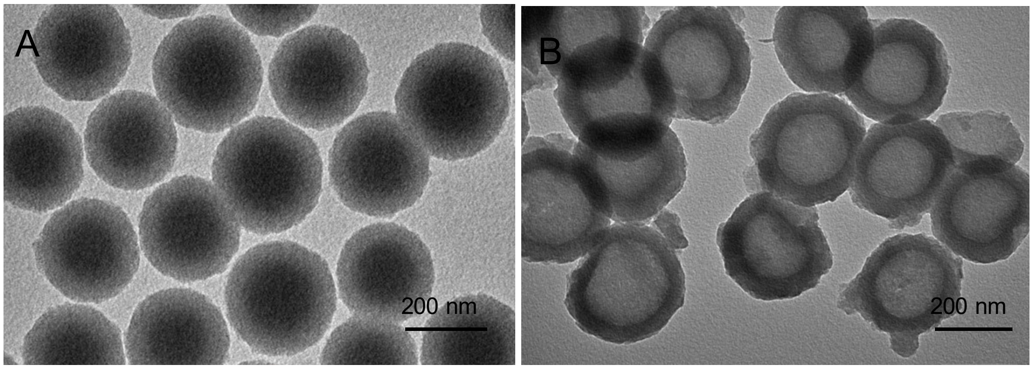


**Figure S1.** TEM images of (A) mesoporous silica nanospheres and (B) HCNs.

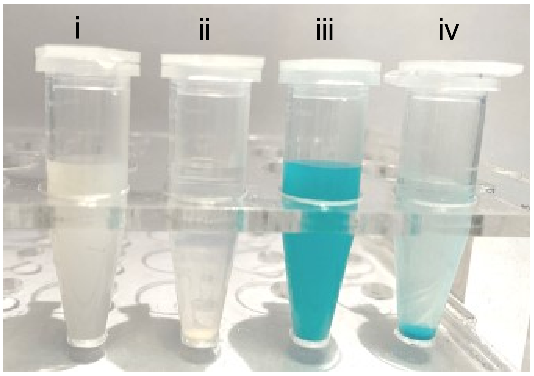


**Figure S2.** Photographs of HCNs suspension (i) before and (ii) after centrifugation and HCNCs suspension (iii) before and (iv) after centrifugation.

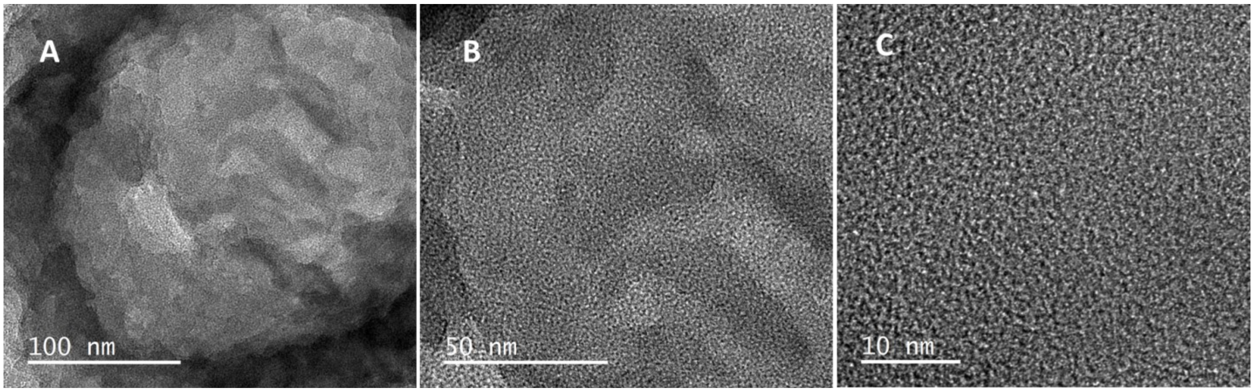


**Figure S3.** HRTEM images of HCNCs.

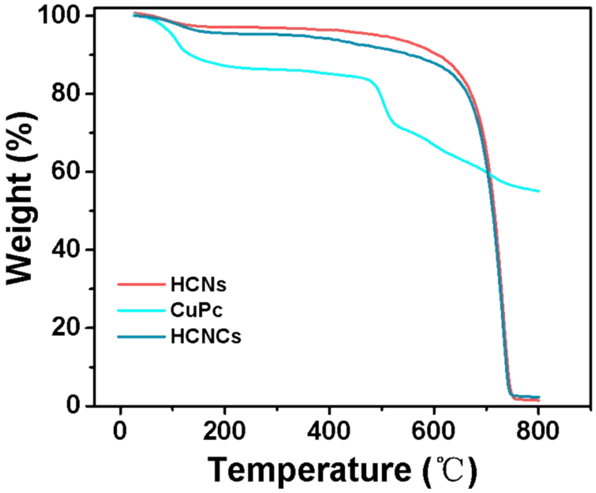


**Figure S4.** The TG curves of CuPc, HCNs and HCNCs. A slight weight loss at 100 °C is observed, which is caused by the physically adsorbed moisture. Then, the second weight losses appear at 480 °C for CuPc and 600 °C for HCNs and HCNCs, which is due to the crumbling of Pc macrocycle and the tri-s-triazine unite.[7,8] This indicates the thermal stability of the CuPc, HCNs and HCNCs during the solvothermal treatment.

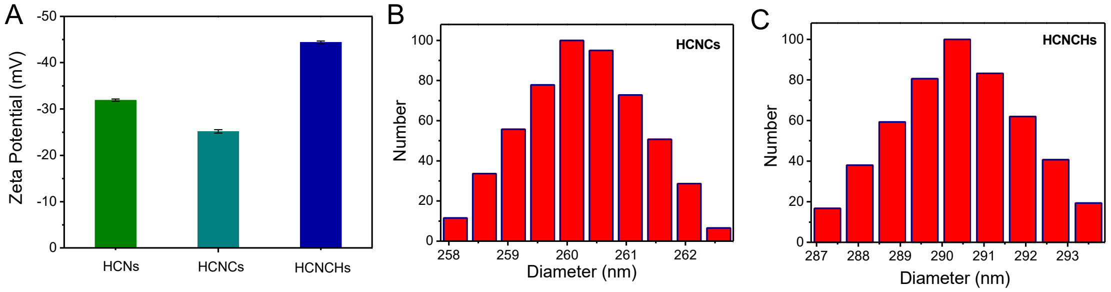


**Figure S5.** (A) ζ-potential evolution of the HCNs, HCNCs and HCNCHs. (B,C) Size distributions of the HCNCs and HCNCHs. The zeta potential decreased from -25.5 mV to -44.4 mV due to the negatively charged carboxy groups on HA, and the average hydrodynamic diameter of HCNCHs increased from 260 nm to 290 nm.

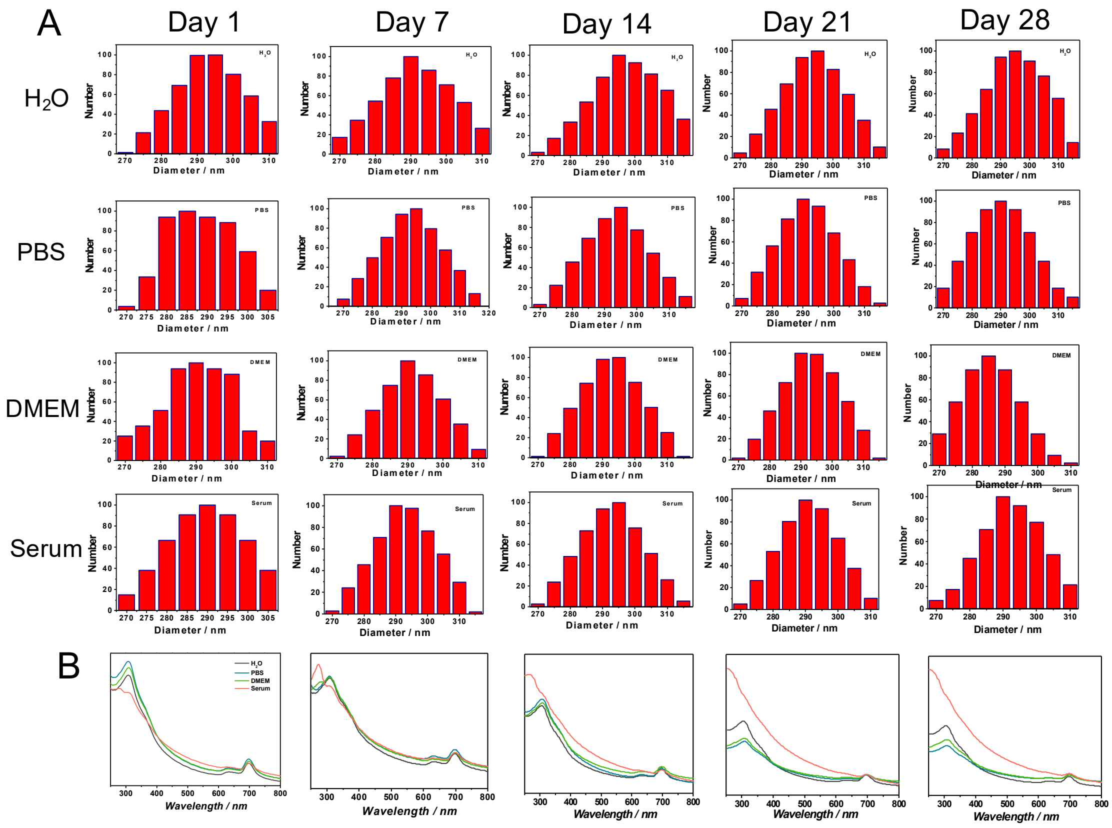


**Figure S6.** (A) DLS, (B) UV-vis analysis of the HCNCHs in $H_2O$, PBS, DMEM and serum with incubation time of 24 h, 7 days, 14 days, 21 days and 28 days.

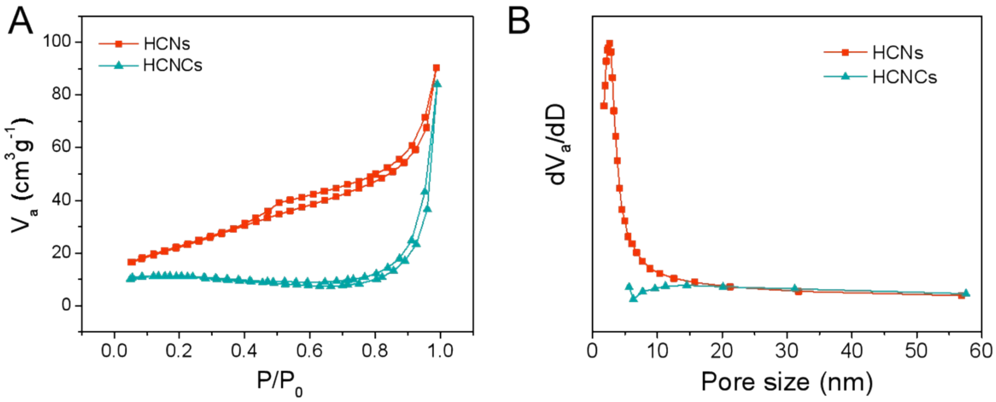


**Figure S7.** (A) $N_2$ adsorption-desorption isotherms and (B) pore size distributions of HCNs and HCNCs. The BET shows mesoporosity for HCNs with surface area of 82 $m^2$ $g^{-1}$ and a uniform pore size distribution of about 2.5 nm. After CuPc loading, the surface area of HCNCs decreases to 30 $m^2$ $g^{-1}$, and the pores mainly distributed near 10 nm. This validates the CuPc grew and filled the mesopores to certain extent, suggesting the loading capacity of the mesoporous nanostructure of HCNs.

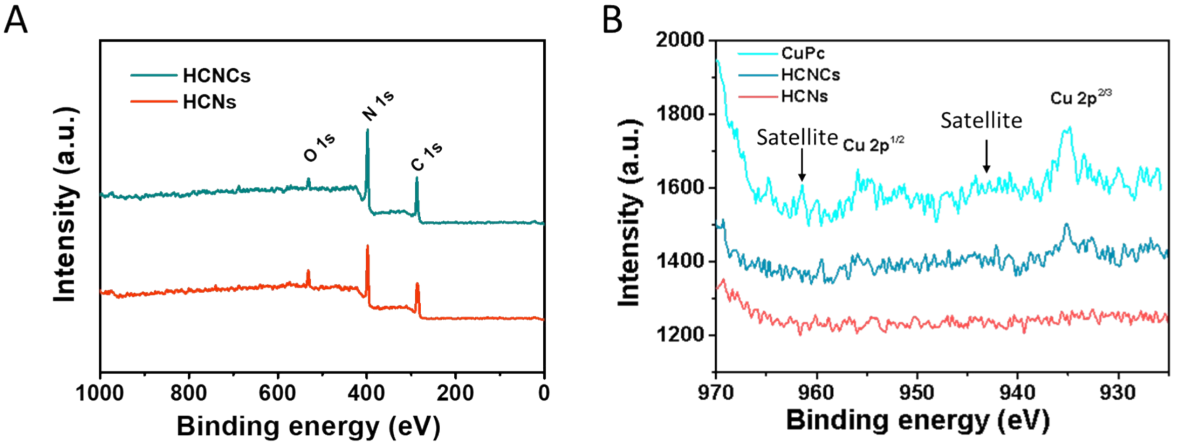


**Figure S8.** (A) XPS spectra of HCNs and HCNCs. (B) High-resolution XPS of CuPc, HCNs and HCNCs. HCNCs shows a higher C and N atomic content than that of HCNs, and there are two

peaks at 954.3 and 934.5 eV in the high-resolution XPS spectra of CuPc and HCNCs, which are corresponding to the Cu $2p^{1/2}$ and Cu $2p^{2/3}$, respectively.[9] Moreover, the two shakeup satellite lines observed at 943.5 and 963.9 eV are characteristic of $Cu^{II}$ with the $d^9$ configuration in the ground state.[10] This validates the successful loading of the CuPc.

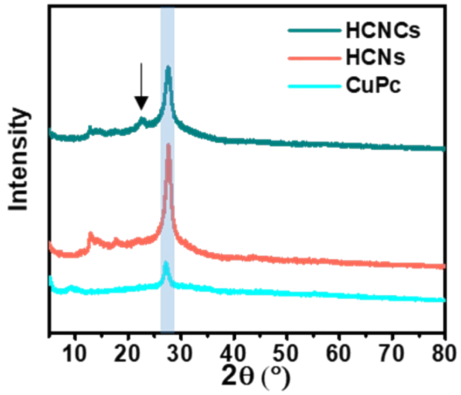


**Figure S9.** XRD spectra of CuPc powder, HCNs and HCNCs.

2. **SERS Study of HCNCs.**

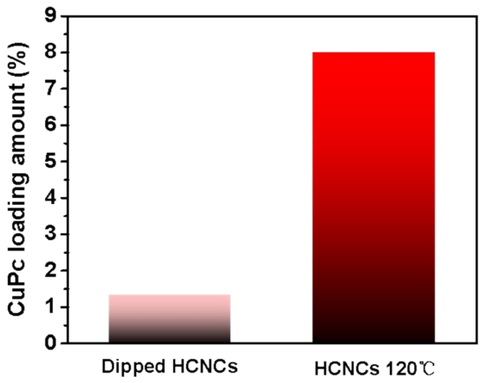


**Figure S10.** CuPc loading percentage of dipped and solvothermal HCNCs. The amount of CuPc on solvothermal HCNCs is 6 times higher than that of the dipped one. This indicates the

increased molecule loading capacity and enhanced substrate-molecule interactions by solvothermal treatment co-contribute to the higher SERS intensity.

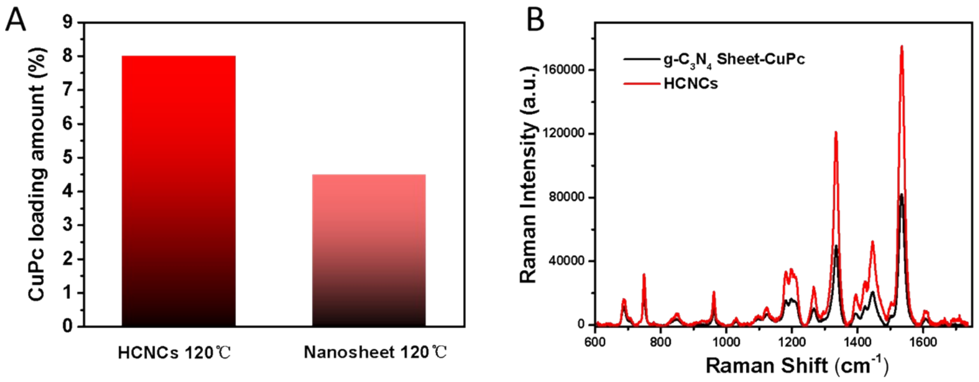


**Figure S11.** (A) CuPc loading percentage and (B) SERS spectra of the solvothermal g-$C_3N_4$ nanosheet-CuPc and HCNCs. The CuPc mass content on the HCNs and g-$C_3N_4$ nanosheet are 8 % and 4.5 %, respectively. This result indicates a 77 % higher molecule loading capacity of the hollow mesoporous structure. The mesoporous HCNs present 135 % higher SERS intensity than that of the nanosheet structure. Here, the SERS intensity enhancement of HCNCs is higher than that of the CuPc loading percentage, suggesting the light-harvesting hollow mesoporous structure contributes to the SERS activity improvement. Therefore, the mesoporous hollow nanostructure is preferable for SERS enhancement because of its molecule loading and unique light-harvesting advantages.

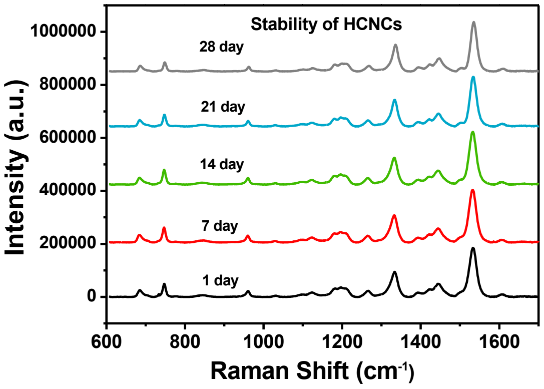


**Figure S12.** SERS stability test of HCNCs during 28 days storage with measurement parameters of 633 nm laser, 1.7 mW power and 10 s exposure time.

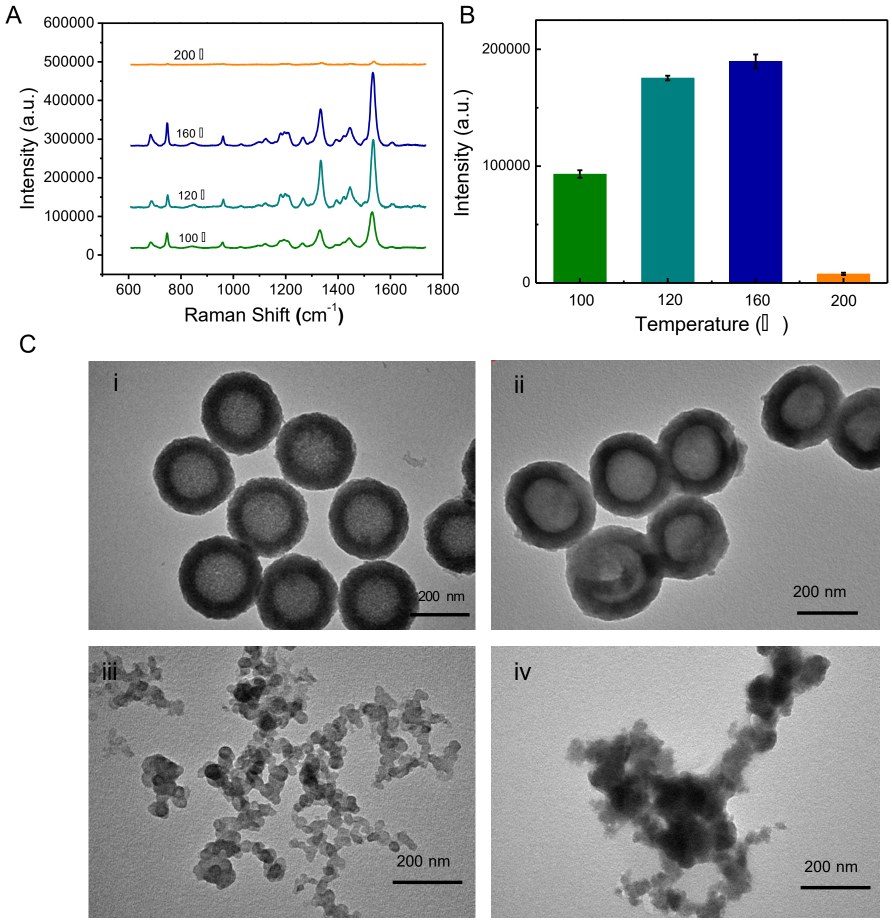

**Figure S13.** (A) SERS spectra, (B) SERS intensity at 1533 $cm^{-1}$ with measurement parameters of 633 nm laser, 1.7 mW power and 10 s exposure time and (C) TEM images of the HCNCs obtained at 100, 120, 160 and 200 °C. The SERS signal increases with the rising T (100, 120, 160 and 200 °C) and reaches the highest level at 160 °C. The signal at 200 °C undergoes a sudden drop. The TEM characterization shows that the mesoporous nanostructure undergoes collapse at 160 and 200°C. Although the SERS intensity at 160 °C is the best, the morphology and dispersibility are perturbed. Thus, 120 °C was chosen as the optimal T for the HCNCs synthesis as the comparatively high intensity and monodispersity for further bioapplication.

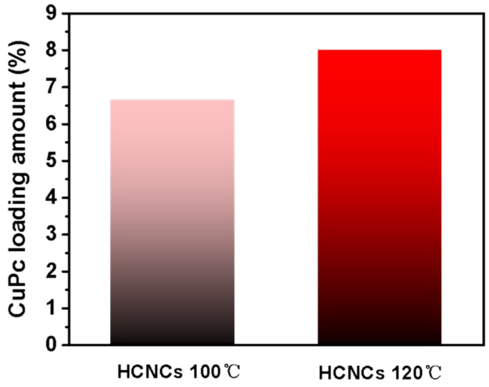


**Figure S14.** CuPc loading percentage of 100 ℃ and 120 ℃ solvothermal HCNCs. The AAS result demonstrates 19 % higher CuPc loading amount of HCNCs-120 ℃ than that of HCNCs-100 ℃. The HCNCs-120 ℃ present 88 % higher SERS intensity than that of the HCNCs-100 ℃. This indicates that the increased molecule loading capacity accompanied by enhanced substrate-molecule interactions under 120 ℃ co-contribute to the higher SERS intensity.

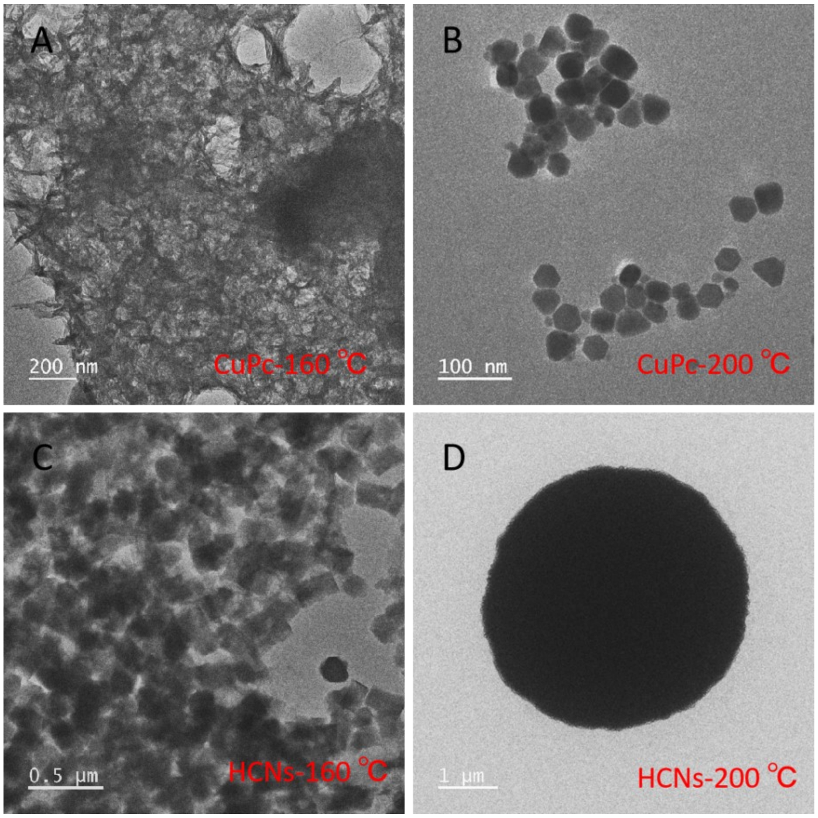


**Figure S15.** TEM images of the solvothermal products of (A) CuPc-160 °C, (B) CuPc-200 °C, (C) HCNs-160 °C, and (D) HCNs-200 °C. The TEM images of CuPc-160 °C and CuPc-200 °C show a tendency of CuPc nanoparticles formation. Similarly, the HCNs-160 °C and HCNs-200 °C undergo a morphology change from nanoscale rod to microscale sphere. This demonstrates the relative high thermal and pressure stress in solvothermal treatment drive the Pc macrocycle and the tri-s-triazine unite to recrystallize through π-π interactions.[11,12] When the CuPc and HCNs were mixed, the recrystallization process also occurs with products of different morphology compared to the single component ones. This can be interpreted by the interference between HCNs and CuPc due to their different chemical structures. Therefore, the recrystallization influences the contact area between the HCNs and CuPc, which results in SERS signal changes at 160 and 200 °C.

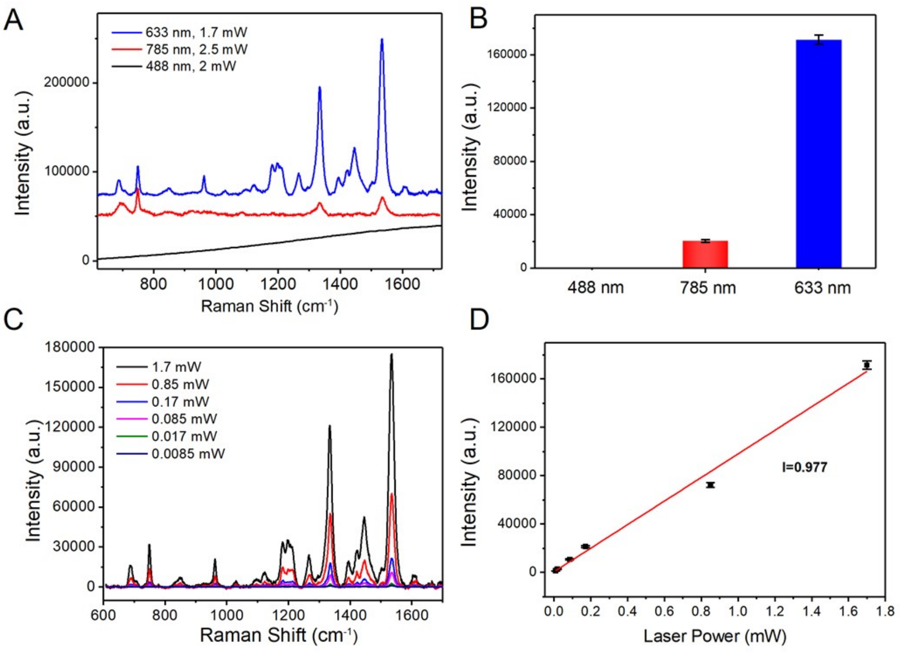


**Figure S16.** (A) SERS spectra of the HCNCs and (B) SERS signal at 1533 $cm^{-1}$ excited by 488, 785 and 633 nm lasers with the excitation power of 2, 2.5 and 1.7 mW, respectively. (C) Integrated SERS spectra of the HCNCs and (D) calibration curve of the signal at 1533 $cm^{-1}$ under different laser powers at 633 nm excitation. The SERS signal collected under 633 nm is 8.5 times higher than that of 785 nm, while 488 nm could not excite the Raman signal of HCNCs, which is attributed to the resonance Raman effect of CuPc at 633 nm. We then observed the SERS activity of the HCNCs under 633 nm laser excitation with different laser powers. The intensity linearly increases as the laser power increases with an achieved detectable power down to 8.5 μW, verifying the stable and high SERS activity of the HCNCs.

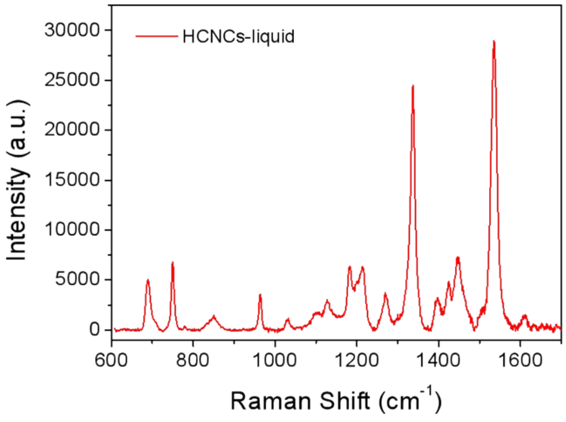


**Figure S17.** Solution-state SERS spectrum of HCNCs with 633 nm laser, 17 mW power and 10 s exposure time.

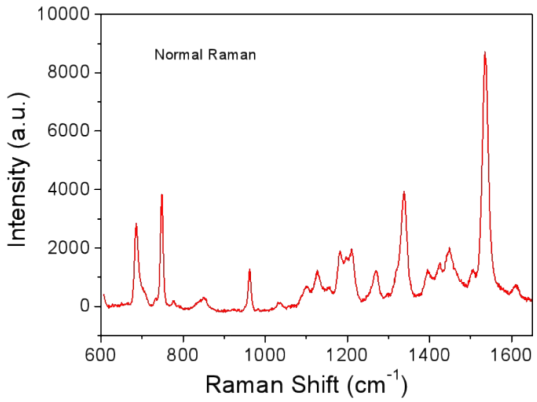


**Figure S18.** Normal Raman spectrum of 0.1 M CuPc with measurement parameters of 633 nm laser, 1.7 mW power and 10 s exposure time.

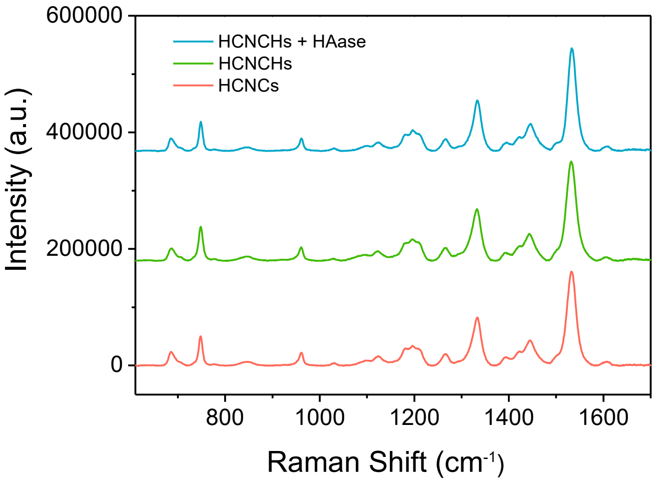


**Figure S19.** SERS spectra of the HCNCs, HCNCHs and HCNCHs treated by HAase with measurement parameters of 633 nm laser, 1.7 mW power and 10 s exposure time.

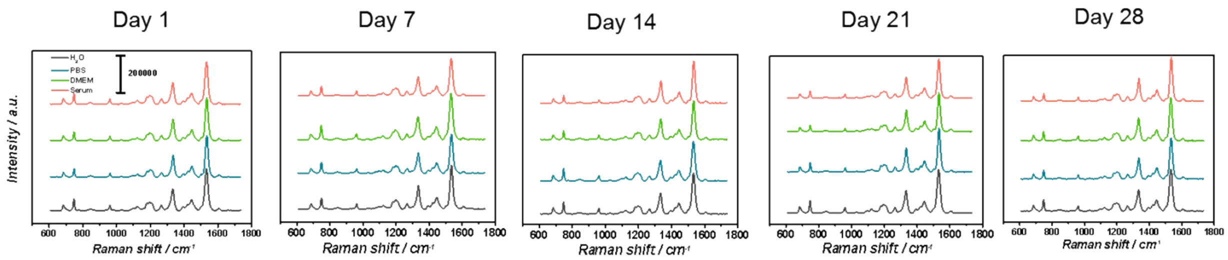


**Figure S20.** SERS analysis of the HCNCHs in $H_2O$, PBS, DMEM and serum with incubation time of 24 h, 7 days, 14 days, 21 days and 28 days.

## 3. Photosynthetic-Mimicking nOER-tPDT Performance of the HCNCs.

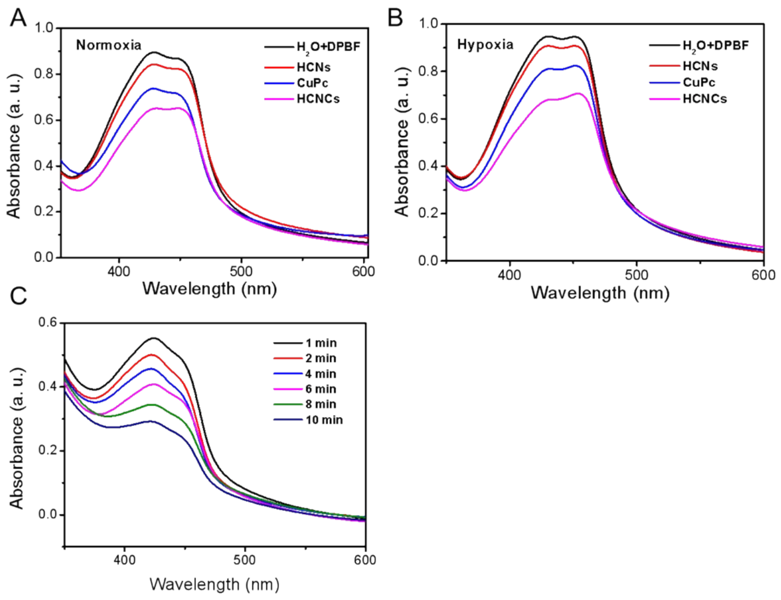


**Figure S21.** UV-vis spectra of 1,3-diphenylisobenzofuran (DPBF) after incubated with HCNs, CuPc and HCNCs under light irradiation (660 nm, 0.282 W $cm^{-2}$ ) for 10 min in (A) normoxia and (B) hypoxia environment. (C) UV-vis spectra of DPBF incubated with HCNCs with increasing laser exposure time.

## 4. Peroxidase-Mimicking Characterization of the HCNCs.

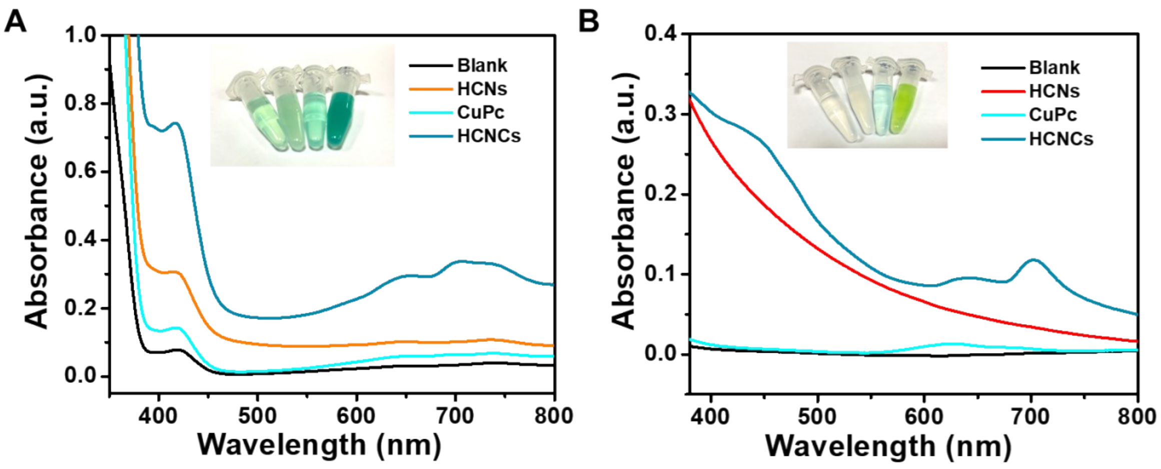


**Figure S22.** UV-vis absorption spectra of (A) oxABTS and (B) oxOPD catalyzed by water, HCNs, CuPc and HCNCs. The inset is the corresponding visual color changes.

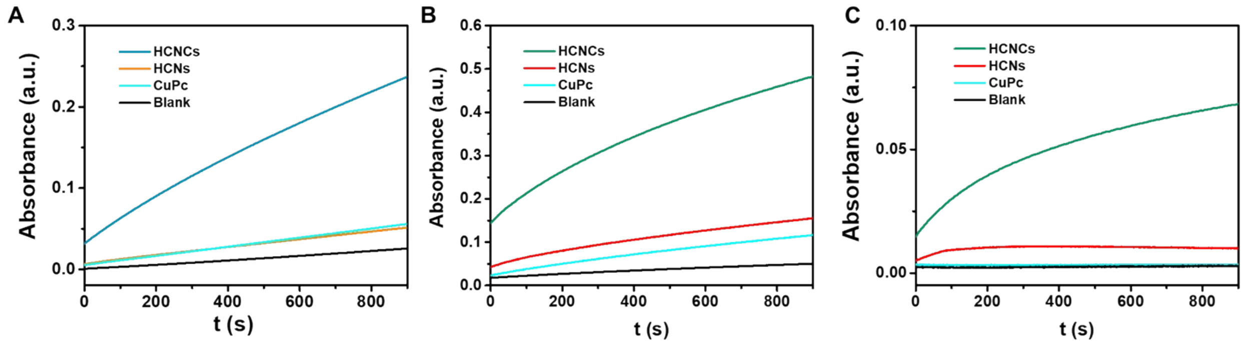


**Figure S23.** Time-dependent absorbance at (A) 653 nm, (B) 420 nm and (C) 450 nm measured from the reaction solutions containing 1 mM TMB, ABTS or OPD, and 10 mM $H_2O_2$ and 100 μg $mL^{-1}$ HCNCs in 0.2 M pH 5.5 HAc/NaAc buffer, 0.2 M pH 4.0 HAc/NaAc buffer, and buffer containing 0.2 M $Na_2HPO_4$ and 0.1 M citric acid, respectively.

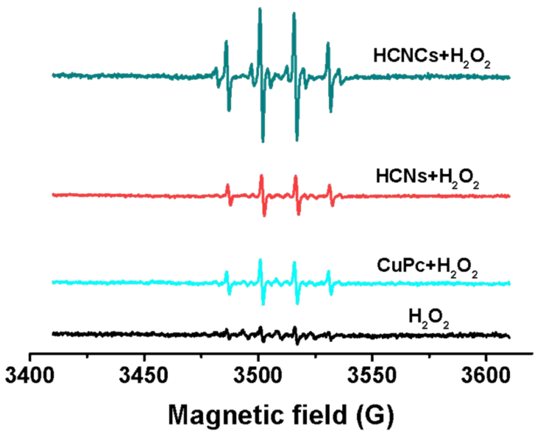


**Figure S24.** ESR spectra of $H_2O_2$, CuPc+$H_2O_2$, HCNs+$H_2O_2$ and HCNCs+$H_2O_2$. 5,5-Dimethyl-1-pyrroline-N-oxide (DMPO) was used as spin adduct.

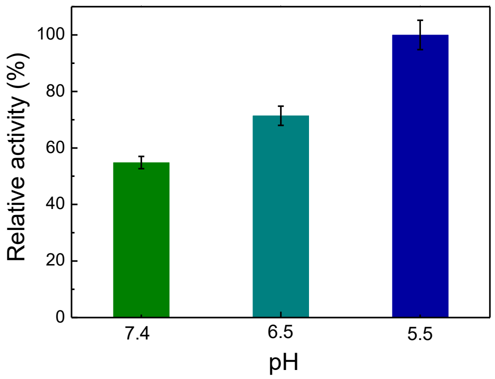


**Figure S25.** The effect of pH on the POD catalytic activity of the HCNCs in 0.2 M HAc/NaAc buffer. Low pH benefits the catalytic activity of HCNCs and considerable catalytic efficiency presents at the tumor physiological pH 6.5.

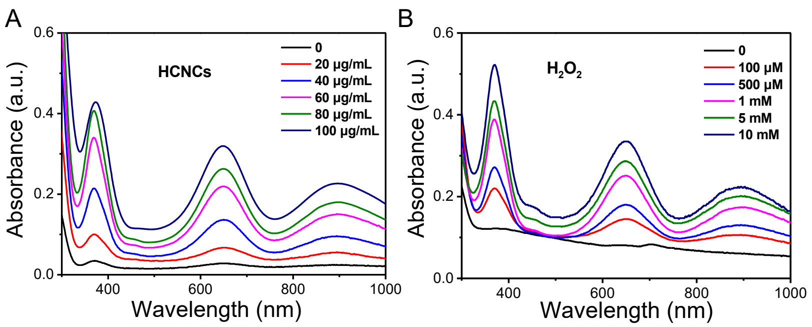


**Figure S26**. (A) HCNCs particle concentration-dependent absorbance at 653 nm measured from the reaction solutions containing 10 mM $H_2O_2$, 1 mM TMB in 0.2 M HAc/NaAc buffer, pH 5.5 at room temperature. (B) $H_2O_2$ concentration-dependent absorbance at 653 nm measured from the reaction solutions containing 100 μg $mL^{-1}$ HCNCs, 1 mM TMB in 0.2 M HAc/NaAc buffer, pH 5.5 at room temperature.

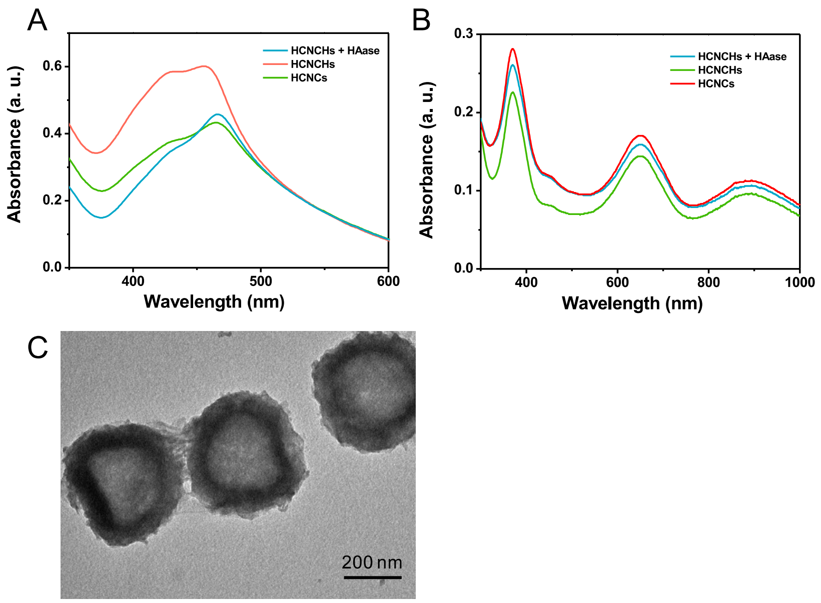


**Figure S27**. (A) PDT and (B) POD activities of HCNCs, HCNCHs and HCNCHs treated by HAase. (C) TEM image of the HAase-treated HCNCHs. The TEM image shows an apparent

collapse of the HA layer on HCNCHs after HAase incubation. Moreover, the tPDT and POD activities of HCNCHs undergo an increasement after HA decomposition, reaching the comparative level of the HCNCs, which suggests a HAase-responsible therapy recovery effect.

## 5. <u>DFT Calculations.</u>

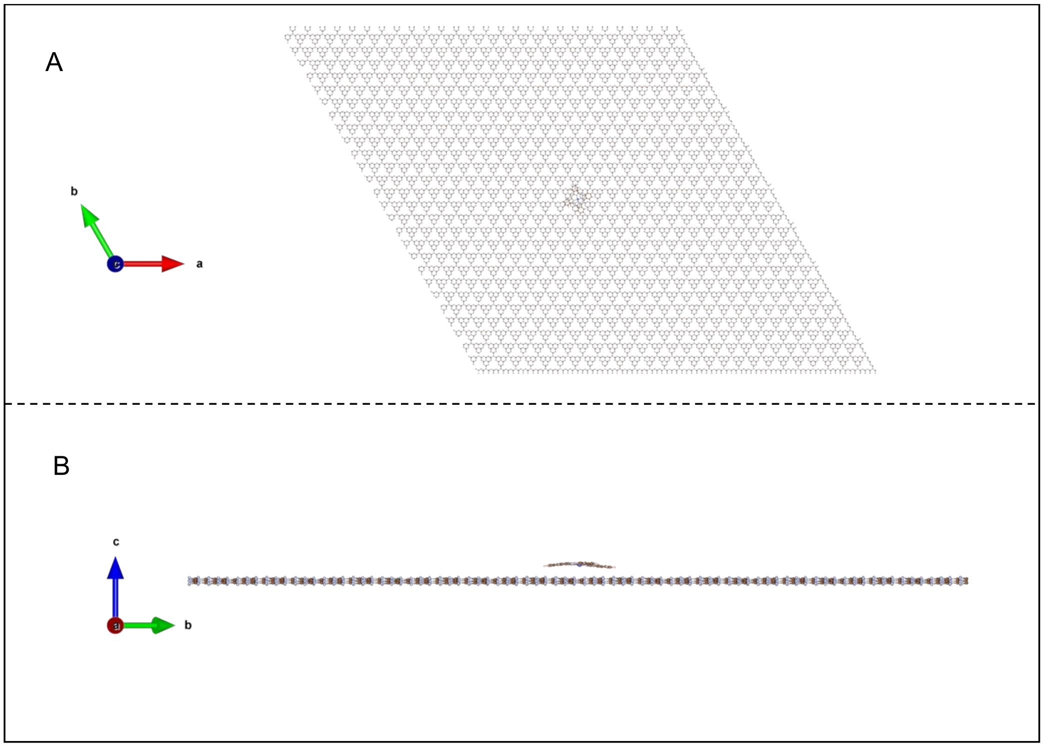


**Figure S28.** Atomic model of the HCNCs used in DFT calculations. (A) Top and (B) side views of the model.

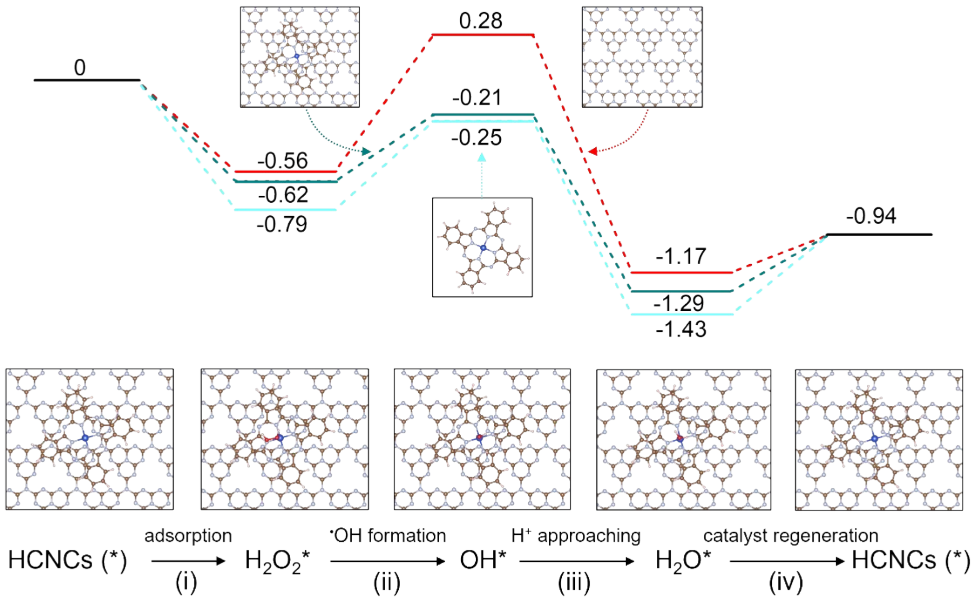


**Figure S29.** The proposed reaction mechanism schematics and free energy diagrams of HCNs, CuPc and HCNCs in heterogeneous POD catalytic reaction towards generating •OH. The heterogeneous peroxidase-mimicking (POD) reaction mechanism on the HCNCs was studied by DFT simulation. The POD-like activity on catalytic surface under acidic conditions involves a four-step process as depicted in the energy profile. The $H_2O_2$ molecule is first adsorbed on the Cu atom of HCNCs (i) with smaller adsorption energy (-0.62 eV) compared with HCNs (-0.56 eV), indicating a stronger attraction of HCNCs for the $H_2O_2$ molecule because of the CuPc modification. Subsequently, the $H_2O_2^*$ is dissociated homogeneously to form a reactive hydroxyl radical (•OH) and a hydroxyl group absorbed on the catalytic surface ($OH^*$) (ii) with energy barriers of 0.41, 0.84 and 0.54 eV for HCNCs, HCNs and CuPc, respectively. Obviously, the energy barrier from this rate-determining step of HCNCs is smaller than that of HCNs and CuPc, demonstrating the homolytic dissociation of the adsorbed $H_2O_2$ entity is more favorable on HCNCs, which thus suggests a stronger POD-like activity. Then, in the acid catalytic environment, a protonated hydrogen atom approaches the $OH^*$ to form an adsorbed $H_2O^*$ on the

HCNCs (iii), which then undergoes desorption from the catalyst surface (iv) that is surmountable at room temperature.[13,14] The desorption of $H_2O$ molecule enables the regeneration of the catalyst surface to activate another $H_2O_2$ for •OH generation in the next cycle.

## 6. Characterization of the HCNCHs and the Intracellular Study.

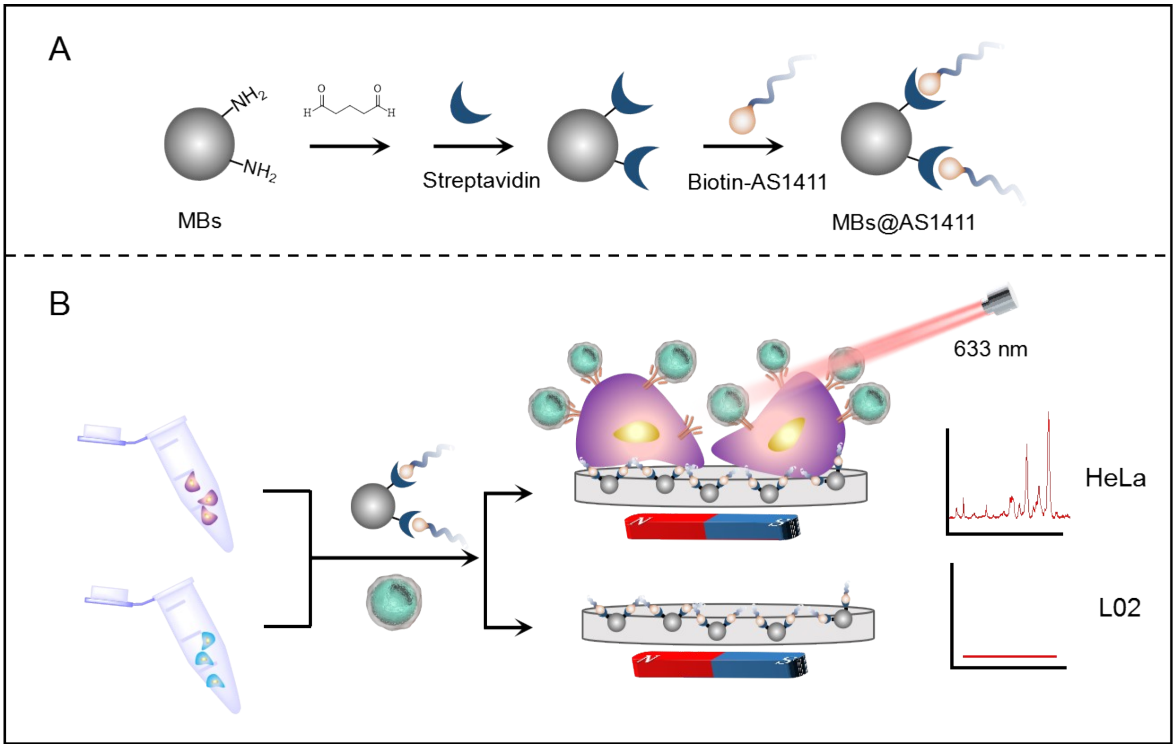


**Scheme S1.** Schematic illustration of (A) the synthesis of the MBs@AS1411 and (B) the SERS capture-identification diagnostic platform construction.

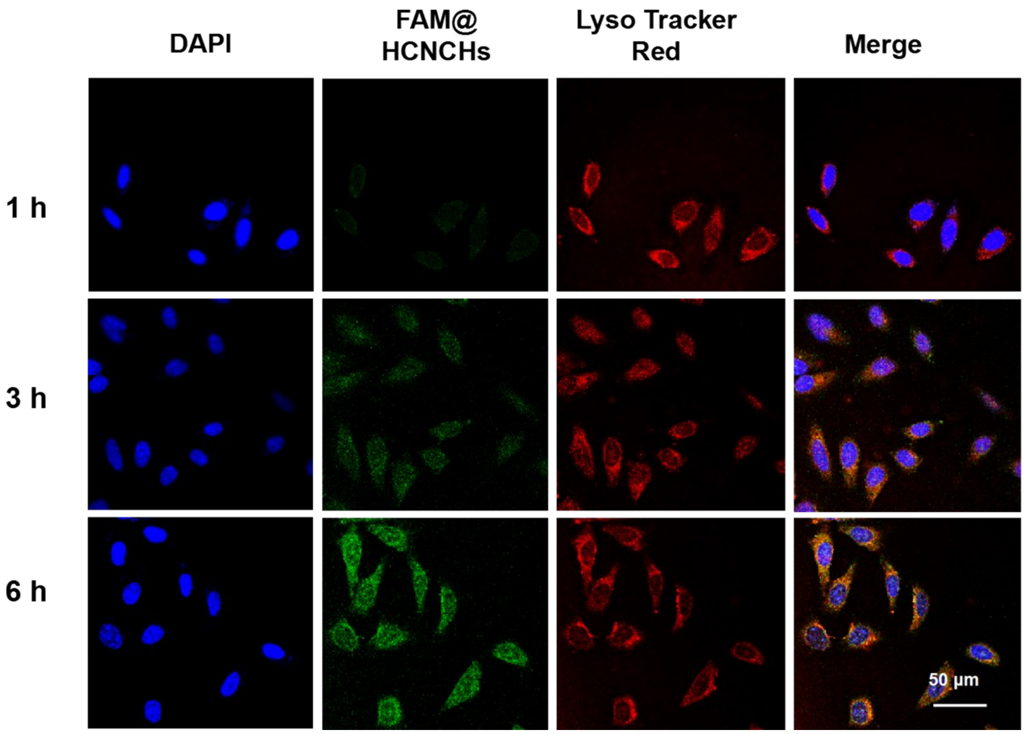


**Figure S30.** The colocalized CLSM imaging of FAM-labeled HCNCHs (green) in the HeLa cells at incubation time point of 6 h. DAPI (blue) and LysoTracker Red (red) are used to stain the cell nucleus and lysosomes, respectively. The CLSM result shows that the green FL signal of FAM-labeled HCNCHs (HCNCHs@FAM) in HeLa cells increased gradually with the prolonging incubation period. Meanwhile, co-localization staining suggests that the HCNCHs were effectively entrapped into the lysosomes and endosomes. Also, the green FL appears at the nuclear region, indicating the HA protective layer of the HCNCHs would decompose in the presence of lysosomal HAase, thereafter releasing the FAM into the nuclear region. This also implies the successful exposure of the effective photo-catalysis/nanozyme interface on HCNCs for further combined therapy.

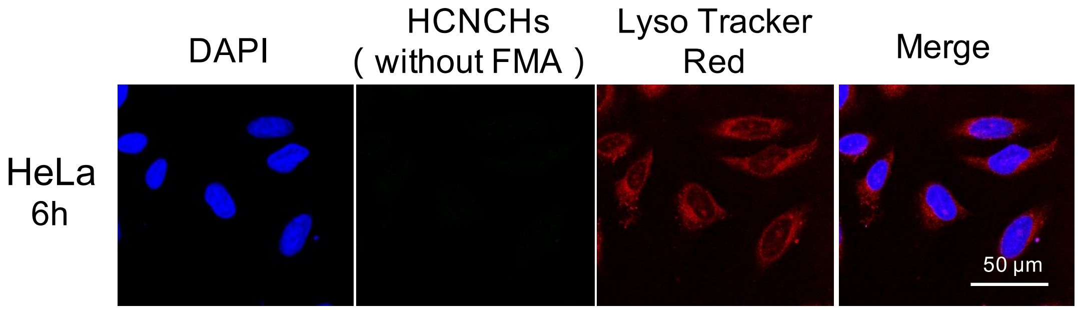


**Figure S31.** The colocalized CLSM imaging of HCNCHs (with no FAM labeling) in the HeLa cells at incubation time point of 6 h. DAPI (blue) and LysoTracker Red (red) are used to stain the cell nucleus and lysosomes, respectively.

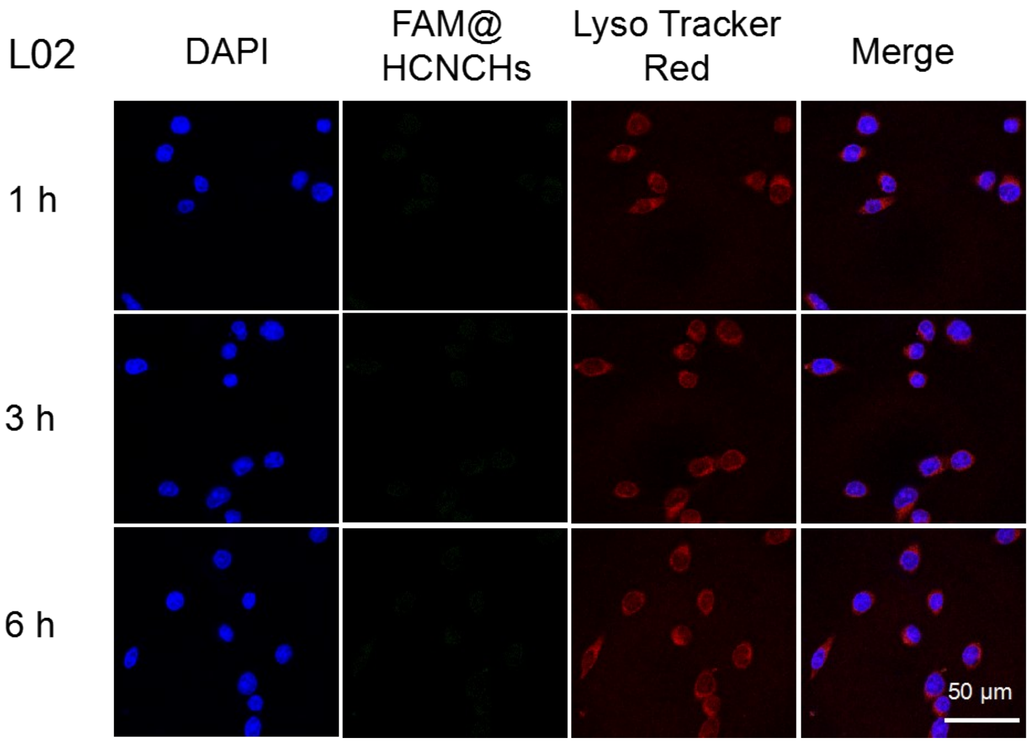


**Figure S32.** The colocalized CLSM imaging of FAM-labeled HCNCHs (green) in the L02 cells at incubation time point of 1, 3, 6 h. DAPI (blue) and LysoTracker Red (red) are used to stain the cell nucleus and lysosomes, respectively. No obvious FL was observed in L02 cells.

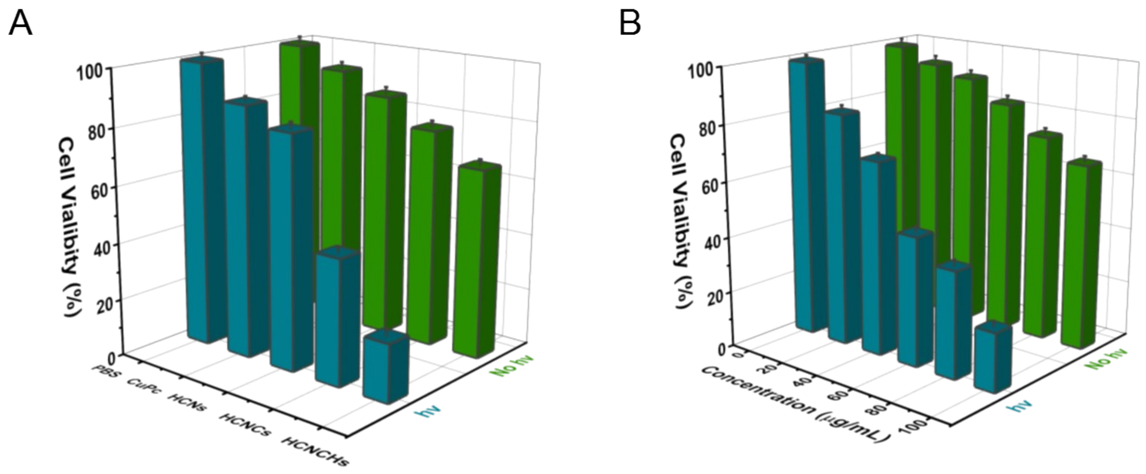


**Figure 33.** Cytotoxicity assay of HeLa cells incubated with (A) PBS, CuPc, HCNs, HCNCs and HCNCHs and (B) different concentration of HCNCHs without and with light irradiation (660 nm, 0.282 W $cm^{-2}$).

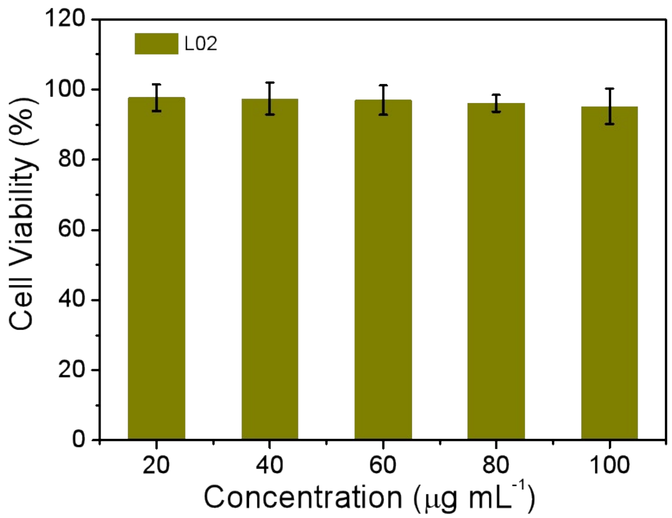


**Figure S34.** Cytotoxicity assay of the HCNCHs to normal L02 cells.

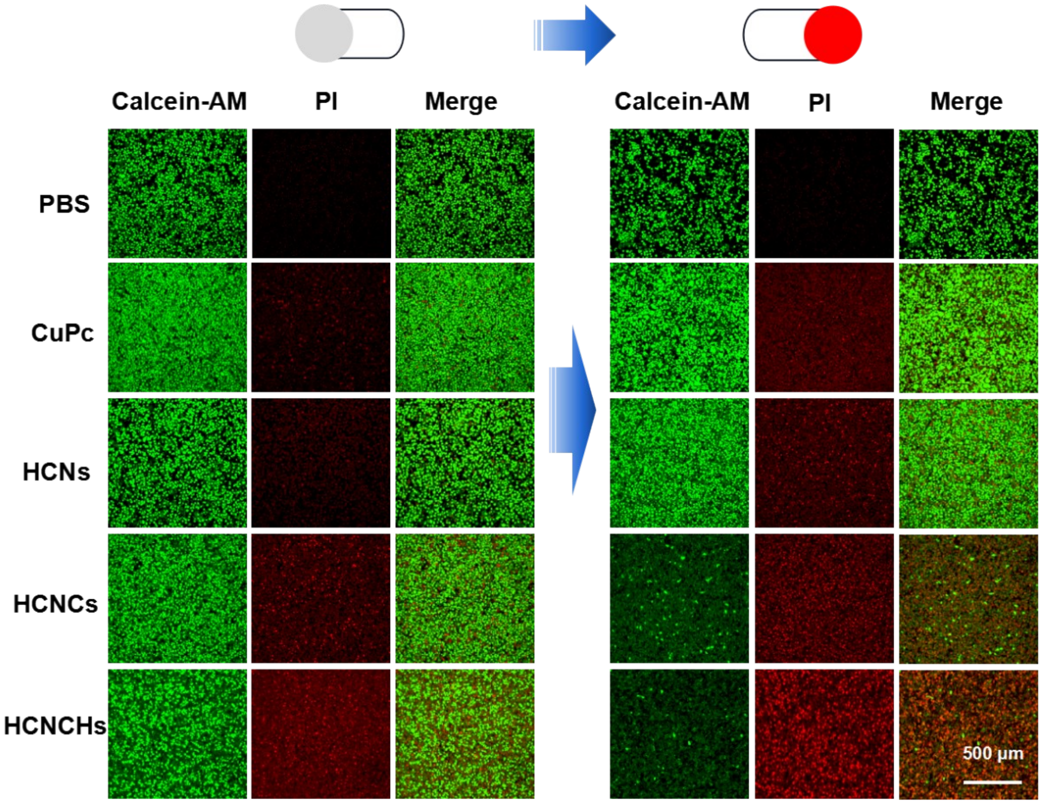


**Figure S35.** Live/dead cell staining assay of HeLa cells treated with PBS, CuPc, HCNs, HCNCs and HCNCHs without and with light irradiation (660 nm, 0.282 W cm$^{-2}$).

## 7. <u>*In Vivo* Assay.</u>

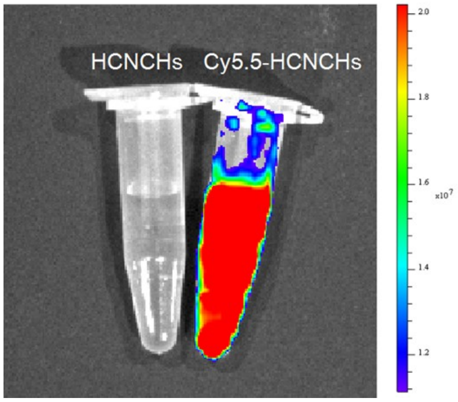


**Figure S36.** FL image of the HCNCHs and Cy5.5-labeled HCNCHs.

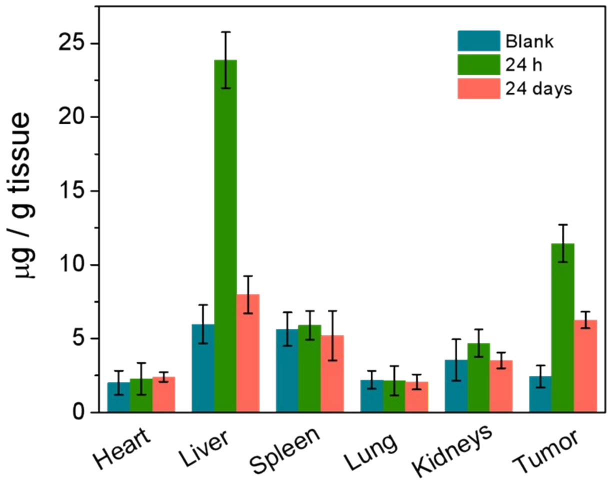


**Figure S37.** ICP-AES analysis of the Cu content in the major organs and tumor after 24 h postinjection and 24 days therapy using HCNCHs.

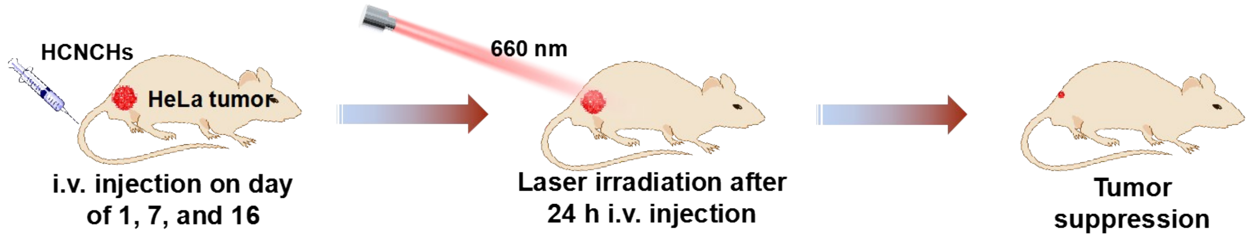


**Scheme S2.** Time schedule of the treatment process.

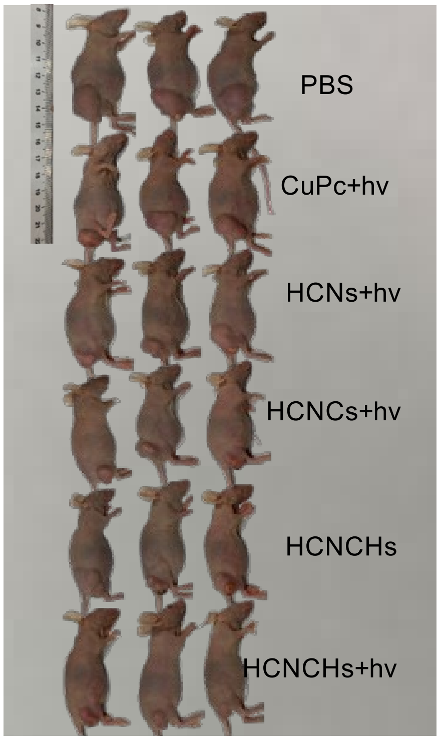


**Figure S38.** Digital photographs of the mice after therapy treatments of PBS, CuPc + light, HCNs + light, HCNCs + light, HCNCHs and HCNCHs + light.

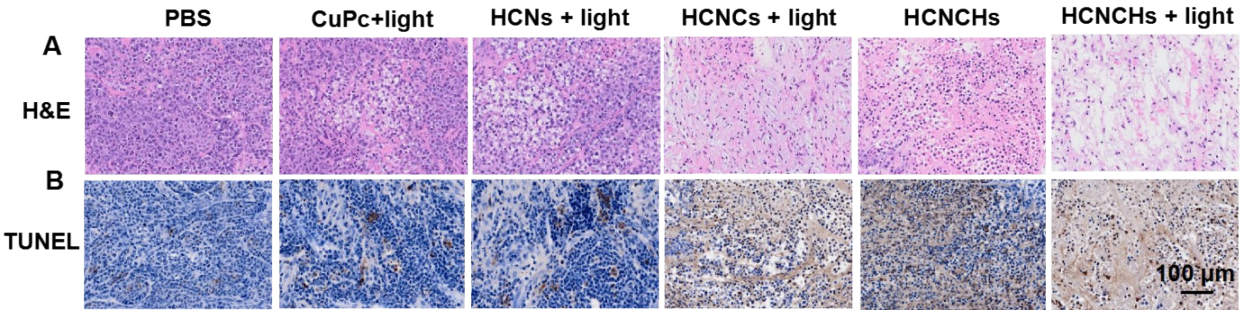


**Figure S39.** (A) Tumor sections H&E and (B) TUNEL staining of mice after treatments of PBS, CuPc + light, HCNs + light, HCNCs + light, HCNCHs and HCNCHs + light.

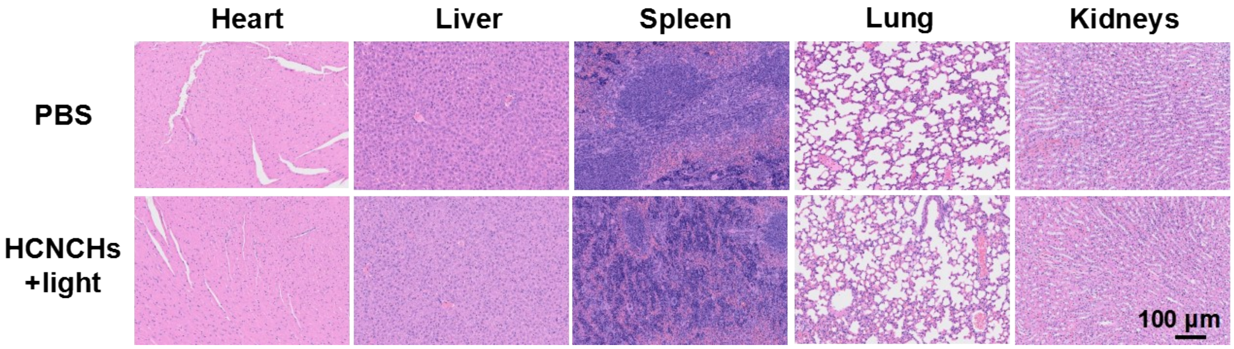


**Figure S40.** Images of H&E stained tissue slices from the major organs (heart, liver, spleen, lung, and kidney) of mice treated by PBS and HCNCHs + light.

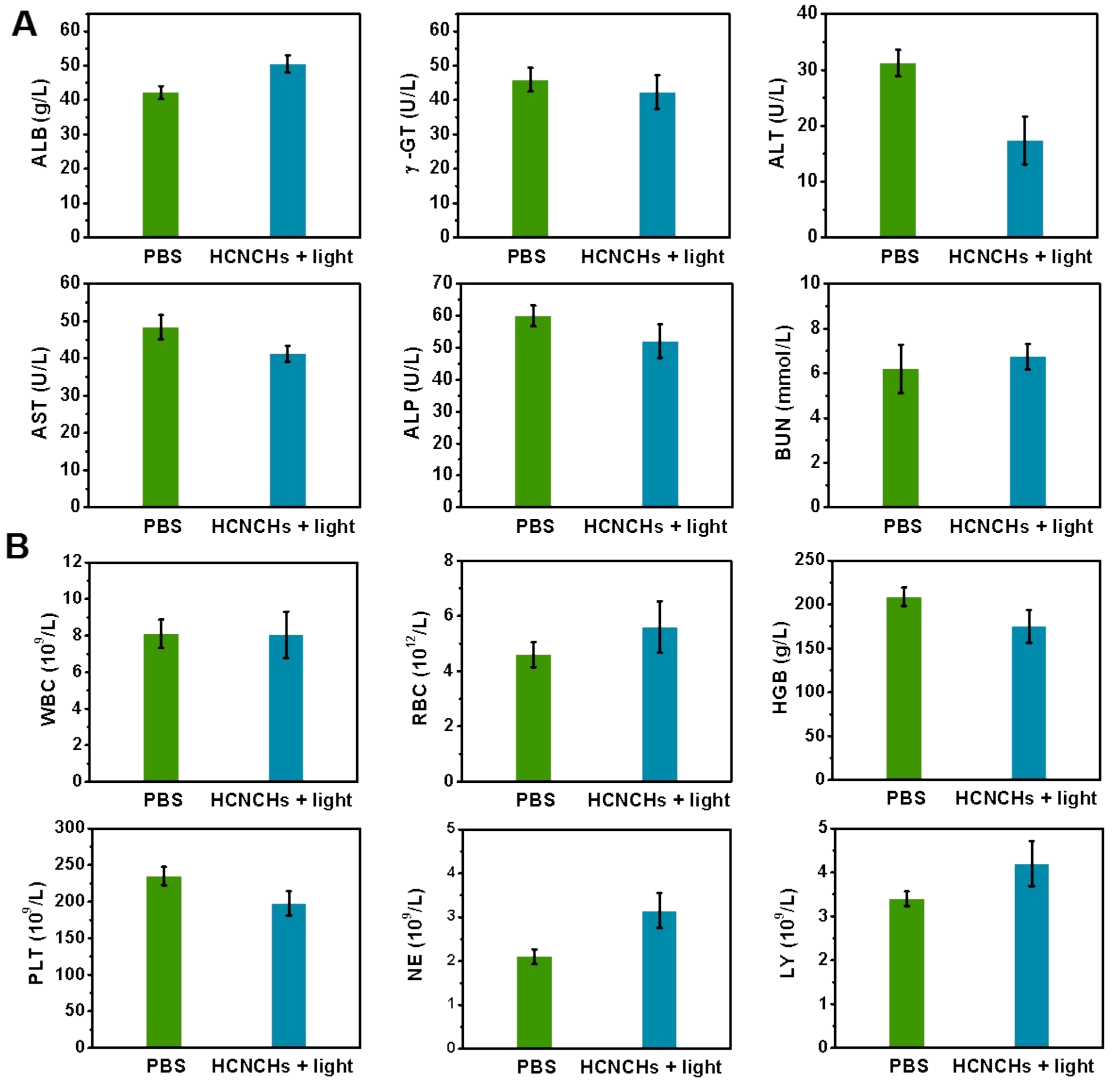


**Figure S41.** (A) Serum biochemical data of PBS and HCNCHs + light-treated mice, including albumin (ALB), γ-glutamyl transpeptidase (γ-GT), alanine aminotransferase (ALT), aspartate aminotransferase (AST), alkaline phosphatase (ALP) and blood urea nitrogen (BUN) levels. (B) Whole blood analysis of PBS and HCNCHs + light-treated mice, including white blood cells count (WBC), red blood cells count (RBC), hemoglobin (HGB), and platelet count (PLT), % of neutrophils (NE) and % of lymphocyte (LY).

## References


(1) J. Sun, J. Zhang, M. Zhang, M. Antonietti, X. Fu and X. Wang, *Nat. Commun.*, 2012, **3**, 1139.

(2) J. P. Perdew, K. Burke, M. Ernzerhof, *Phys. Rev. Lett.,* 1996, **77**, 3865-3868.

(3) B. Hammer, L. B. Hansen, J. K. Nørskov, *Phys. Rev. B,* 1999, **59**, 7413-7421.

(4) P. E. Blöchl, *Phys. Rev. B,* 1994, **50**, 17953-17979.

(5) G. Kresse, D. Joubert, *Phys. Rev. B,* 1999, **59**, 1758-1775.

(6) H. J. Monkhorst, J. D. Pack, *Phys. Rev. B,* 1976, **13**, 5188-5192.

(7) Y. S. Korostei, A. Y. Tolbin, A. V. Dzuban, V. E. Pushkarev, M. V. Sedova, S. S. Maklakov and L. G. Tomilova, *Dyes Pigm.*, 2018, **149**, 201-211.

(8) Y. Wang, J. Yu, W. Peng, J. Tian and C. Yang, *Sci. Rep.*, 2019, **9**, 5932.

(9) Y. Li, M. Yang, Z. Tian, N. Luo, Y. Li, H. Zhang, A. Zhou and S. Xiong, *Front. Chem.*, 2019, **7**, 334.

(10) J. Ghijsen, L. H. Tjeng, J. van Elp, H. Eskes, J. Westerink, G. A. Sawatzky, Electronic structure of $Cu_2O$ and CuO, *Physical Review B* **1988,** *38,* 11322-11330.

(11) L. Cui, G. Lv, Z. Dou and X. He, *Electrochim. Acta*, 2013, **106**, 272-278.

(12) B. Zhang, Y. Wang, M. Li, L. Cui and X. He, *J. Electroanal. Chem.*, 2015, **743**, 10-17.

(13) G. Fang, W. Li, X. Shen, J. M. Perez-Aguilar, Y. Chong, X. Gao, Z. Chai, C. Chen, C. Ge and R. Zhou, *Nat. Commun.*, 2018, **9**, 129.

(14) M. Huo, L. Wang, Y. Wang, Y. Chen and J. Shi, *ACS Nano*, 2019, **13**, 2643-2653.
